# Efficacy and safety of sublingual versus subcutaneous immunotherapy in children with allergic rhinitis: a systematic review and meta-analysis

**DOI:** 10.3389/fimmu.2023.1274241

**Published:** 2023-12-15

**Authors:** Jiumei Yang, Sihong Lei

**Affiliations:** The Department of Otolaryngology-Head and Neck Surgery, Nanchong Central Hospital, Second Clinical Medical College of North Sichuan Medical College, Nanchong, Sichuan, China

**Keywords:** subcutaneous immunotherapy, sublingual immunotherapy, allergic rhinitis, efficacy, safety, meta-analysis

## Abstract

**Aim:**

To systematically compare the efficacy and safety of subcutaneous immunotherapy (SCIT) and sublingual immunotherapy (SLIT) in children with allergic rhinitis (AR).

**Methods:**

PubMed, Embase, Cochrane Library, and Web of Science were searched from inception to March 2, 2023. Outcomes included symptom scores (SSs), medication scores (MSs), symptom and medication scores (SMSs), new sensitizations, development of asthma, improvement, and treatment-related adverse events (TRAEs). The quality of the included studies was assessed by the modified Jadad scale and Newcastle-Ottawa scale (NOS). Meta-regression was carried out to explore the source of heterogeneity. Subgroup analysis was further conducted in terms of study design [randomized controlled trials (RCTs), cohort studies], allergen [house dust mites (HDMs), grass pollen], treatment duration (≥ 24, 12-23 or < 12 months), allergen immunotherapy (AIT) modality (drops or tablets), and AIT protocol [continuous, pre-seasonal, co-seasonal, or after the grass pollen season (GPS)]. Sensitivity analysis was conducted for all outcomes. A Bayesian framework and a Monte Carlo Markov Chain (MCMC) model were developed for indirect comparison.

**Results:**

Totally 50 studies with 10813 AR children were included, with 4122 treated with SLIT, 1852 treated with SCIT, and 4839 treated with non-SLIT or non-SCIT therapy. For direct comparison, the SLIT group had a similar SS to the SCIT group [pooled standardized mean difference (SMD): 0.41, 95% confidence interval (CI): -0.46, 1.28, *P* = 0.353]. Comparable MSs were observed in the SLIT and SCIT groups (pooled SMD: 0.82, 95%CI: -0.88, 2.53, *P* = 0.344). For indirect comparison, no significant differences were found in SSs (pooled SMD: 1.20, 95% credibility interval (CrI): -1.70, 4.10), MSs (pooled SMD: 0.57, 95%CrI: -1.20, 2.30), SMSs (pooled SMD: 1.80, 95%CrI: -0.005, 3.60), new sensitizations [pooled relative risk (RR): 0.34, 95%CrI: 0.03, 3.58], and development of asthma (pooled RR: 0.68, 95%CrI: 0.01, 26.33) between the SLIT and SCIT groups; the SLIT group illustrated a significantly lower incidence of TRAEs than the SCIT group (pooled RR: 0.17, 95%CrI: 0.11, 0.26).

**Conclusion:**

Considering both efficacy and safety, SLIT might be a more favorable AIT than SCIT in the treatment of pediatric AR, which may serve as a decision-making reference for clinicians.

**Systematic review registration:**

PROSPERO (CRD42023460693).

## Introduction

Allergic rhinitis (AR), an upper airway disease, is a health concern worldwide, with growing prevalence in the world (1, 2). It affects up to 50% of the global population (3), and often develops in children and adolescents (4). Typical symptoms comprise sneezing, runny nose, itchy nose, and nasal obstruction (5). This disorder influences the quality of life of patients and is related to severe comorbidities such as asthma, sinusitis and conjunctivitis, thus leading to a huge health burden (6). AR was also associated with great economic costs via impacts on education, productivity, and medical resources (7).

Pharmacotherapy is still the standard care for AR treatment (8). When pharmacotherapy is ineffective, allergen immunotherapy (AIT), as a major disease-modifying method, should be taken into account (9). AIT had a long-term disease-modifying effect, and can be administered through a subcutaneous (SCIT) or sublingual (SLIT) route (10). SLIT has been developed as a potential alternative to SCIT (11). Both routes are demonstrated to be clinically effective and safe by existing evidence (12, 13). Nevertheless, most current studies have assessed SCIT or SLIT respectively (14–17), and there is a paucity of studies on the direct comparison of these two routes (18, 19). A previous meta-analysis evaluated the effectiveness and adverse events of SCIT and SLIT in seasonal AR among both children and adults (20). Kim et al. (21) conducted a network meta-analysis to compare the efficacy of SCIT and SLIT for pediatric and adult patients with house dust mite allergy-related AR. A recent meta-analysis investigated the roles of SCIT and SLIT for adults with AR using indirect comparison (22). At present, no meta-analysis of SCIT versus SLIT has been performed specifically for AR children, which necessitates comprehensive research to facilitate AR management in the pediatric population.

This study intended to evaluate and compare the efficacy and safety of SCIT and SLIT in children with AR via a meta-analysis using direct and indirect comparisons, in order to provide a reference for clinical decision-making between SCIT and SLIT.

## Methods

### Search strategy

A comprehensive search was independently conducted by two investigators (JM Yang, SH Lei) on the following four databases from inception to March 2, 2023: PubMed, Embase, Cochrane Library, and Web of Science. Disagreements were settled via discussion. The English search terms were “Rhinitis, Allergic” OR “Allergic Rhinitides” OR “Allergic Rhinitis” OR “Pollen Allerg*” OR “Pollinos*” OR “Hay Fever” OR “Hayfever Rhinoconjunctivitis” OR “Rhinitis” OR “Rhinitides” OR “Dust Mite Allergy” OR “Dust Mite Hypersensitivit*” OR “Dermatophagoides pteronyssinus Allerg*” OR “Dermatophagoides farinae Allerg*” AND “Sublingual immunotherap*” OR “SLIT” OR “Subcutaneous immunotherap*” OR “SCIT” OR “Allergen immunotherapy” OR “Immunotherap*” OR “Immunologic Desensitization” OR “Hyposensitization Therapy” OR “Allergy Shot” OR “Immunosuppression Therap*” OR “Anti Rejection Therap*” OR “Antirejection Therap*” OR “Immunosuppressive Therap*” OR “Immunosuppression*” AND “Child” OR “Children” OR “Pediatric” OR “Pediatrics” OR “Childhood” OR “Adolescen*” OR “Teenager*” OR “Teen” OR “Teens” OR “Youth” OR “Youths”. The retrieved studies were first screened via titles and abstracts, and subsequently via full texts. This systematic review and meta-analysis was carried out following the Preferred Reporting Items for Systematic Reviews and Meta-analyses (PRISMA), and was registered in PROSPERO (registration number: CRD42023460693).

### Inclusion and exclusion criteria

The inclusion criteria included (a) population: studies on children with AR (aged ≤ 18 years); (b) intervention and comparator: studies with SLIT versus non-SLIT, SCIT versus non-SCIT, or SLIT versus SCIT; (c) outcome: studies with any of the following outcomes: symptom scores (SSs), medication scores (MSs), symptom and medication scores (SMSs), new sensitizations, development of asthma, improvement, and treatment-related adverse events (TRAEs); and (d) study design: open or blind controlled trials, cohort studies, or case-control studies.

The exclusion criteria included (a) studies on mixed population, such as patients of all ages or patients with AR or asthma; (b) studies with incomplete data or of which data could not be extracted; (c) meta-analyses, reviews, conference abstracts, animal tests, case reports; or (d) non-English studies.

### Outcome measures

The outcomes in this analysis included SSs, MSs, SMSs, new sensitizations, development of asthma, improvement, and TRAEs. Concerning SSs, the higher the SS, the more severe the symptom (23). Symptoms included itchy nose, sneezing, running nose, blocked nose, itchy eyes, etc. (17, 24, 25), and the Visual Analogue Scale (VAS) was also used for evaluation (15, 26). Some studies recorded daily values (25, 27), while some recorded scoring results over a period of time (28, 29). For MSs, the higher the MS, the more medication was used (14). Some studies recorded average daily dosages (27, 30), while some recorded dosages over a period of time (28, 29). The SMS referred to the combination of the SS and MS.

Improvement was defined as “slight to moderate improvement” and “marked improvement” (31), improvement rates of 26–65% and ≥ 66% based on a scale of 1 to 3 (30), a reduction of over 1 point for symptoms (32), overall treatment effect (33), or self-reported clinical improvement (19).

### Data collection and quality assessment

Two independent investigators (JM Yang, SH Lei) collected the following data from eligible studies: first author, year of publication, country, study design, AR diagnosis, group, group division, treatment, sample size, sex (male/female), age (years), duration of AR (years), allergen, mono-/poly-sensitization status, AIT modality, AIT protocol, product type/name (manufacturer), comorbidity, treatment duration (months), dropout rate (%), quality assessment (QA), and outcome.

The quality of randomized controlled trials (RCTs) was assessed by the modified Jadad scale from random sequence generation, randomization concealment, blinding, and withdrawal and dropout, which had a total score of 7, with 1-3 as low quality and 4-7 as high quality (34). The quality of cohort studies was estimated using the Newcastle-Ottawa scale (NOS) from population selection, intergroup comparability, and result measurement, which had a total score of 9, with 0-3 as poor quality, 4-6 as fair quality, and 7-9 as good quality (35).

### Statistical analysis

Statistical analysis was performed using the Gemtc 1.0.1 package from Stata 15.1 (Stata Corporation, College Station, TX, USA) and R 4.1.3 (R Foundation for Statistical Computing, Vienna, Austria). Measurement data were reported as standardized mean differences (SMDs) and 95% confidence intervals (CIs). When different studies utilize different rating instruments or different measurement units for the same outcome, standardized mean differences (SMDs) can be used in such cases (36). In this study, SMDs were used to handle variations in SS scales or individual SSs even for the same indicators among different studies. Counting data were shown as relative risks (RRs) and 95%CIs. Heterogeneity tests were conducted for each outcome. If the heterogeneity statistic I^2^ ≥ 50%, the random-effects model was used for analysis, and otherwise, the fixed-effects model was applied. Meta-regression was carried out to explore the source of heterogeneity. Subgroup analysis was further conducted in terms of study design (RCTs, cohort studies), allergen [house dust mites (HDMs), grass pollen], treatment duration (≥ 24, 12-23 or < 12 months), AIT modality (drops or tablets), and AIT protocol [continuous, pre-seasonal, co-seasonal, or after the grass pollen season (GPS)]. Sensitivity analysis was conducted for all outcomes by deleting one study at a time and comprehensively assessing the remaining studies.

Indirect comparison refers to indirectly obtaining the relative effect of A versus B through the results of A versus C and B versus C, with C as a common comparator (37). The indirect comparison of A and B is provided by the direct comparison of A and C and the direct comparison of B versus C with (38). The distinction between direct and indirect comparisons is that the direct comparison (i.e. head-to-head comparison) of A and B is directly provided by A versus B trials. The rationales for choosing indirect comparison are as follows: first, there are no studies for the direct comparison of A and B, but each is compared with a common comparator (e.g. C); second, there are studies for direct comparison, but the number or quality of these studies is relatively small or low. The most essential difference between the frequentist method and the Bayesian method lies in their different interpretations of probability. The Bayesian method has a prior distribution, and it treats unknown parameters as random variables, while frequentist statistics treat them as fixed but unknown values. The Bayesian inference allows the probability to be associated with an unknown parameter; the Bayesian interpretation also allows researchers to maintain their own understanding of specific parameter settings; the Bayesian result can be a posterior probability distribution obtained from experiments or research regarding parameters. The conclusion of frequency statistics is to accept or reject hypothesis testing or to see whether the results are included in the confidence interval under a certain sample inference (39). Compared with the frequentist analysis, the Bayesian analysis has the following advantages: (1) the Bayesian approach can not only effectively integrate data and flexibly build models, but also use the obtained posterior probability to rank all interventions participating in the comparison and distinguish comparative advantages and disadvantages, while the frequentist method can only rely on the effect size and its 95%CI obtained by pairwise comparison in ranking; (2) since the frequentist approach uses the maximum likelihood method in parameter estimation, which estimates the maximum likelihood function through continuous iteration, it is prone to instability and biased results, while the Bayesian approach does not have this problem, so its estimated values are more accurate than those of the frequentist approach (39). Then the data were converted into a relative data format. A Bayesian framework and a Monte Carlo Markov Chain (MCMC) model were developed for indirect comparison, the model has a chain number of 4, an initial iteration number of 20000, and a further updated iteration number of 50000, with a step size of 1. Indirect effect sizes and 95% credibility intervals (CrIs) were reported for different outcomes. The difference was statistically significant when *P*<0.05.

## Results

### Characteristics of the included studies

**Figure 1:**
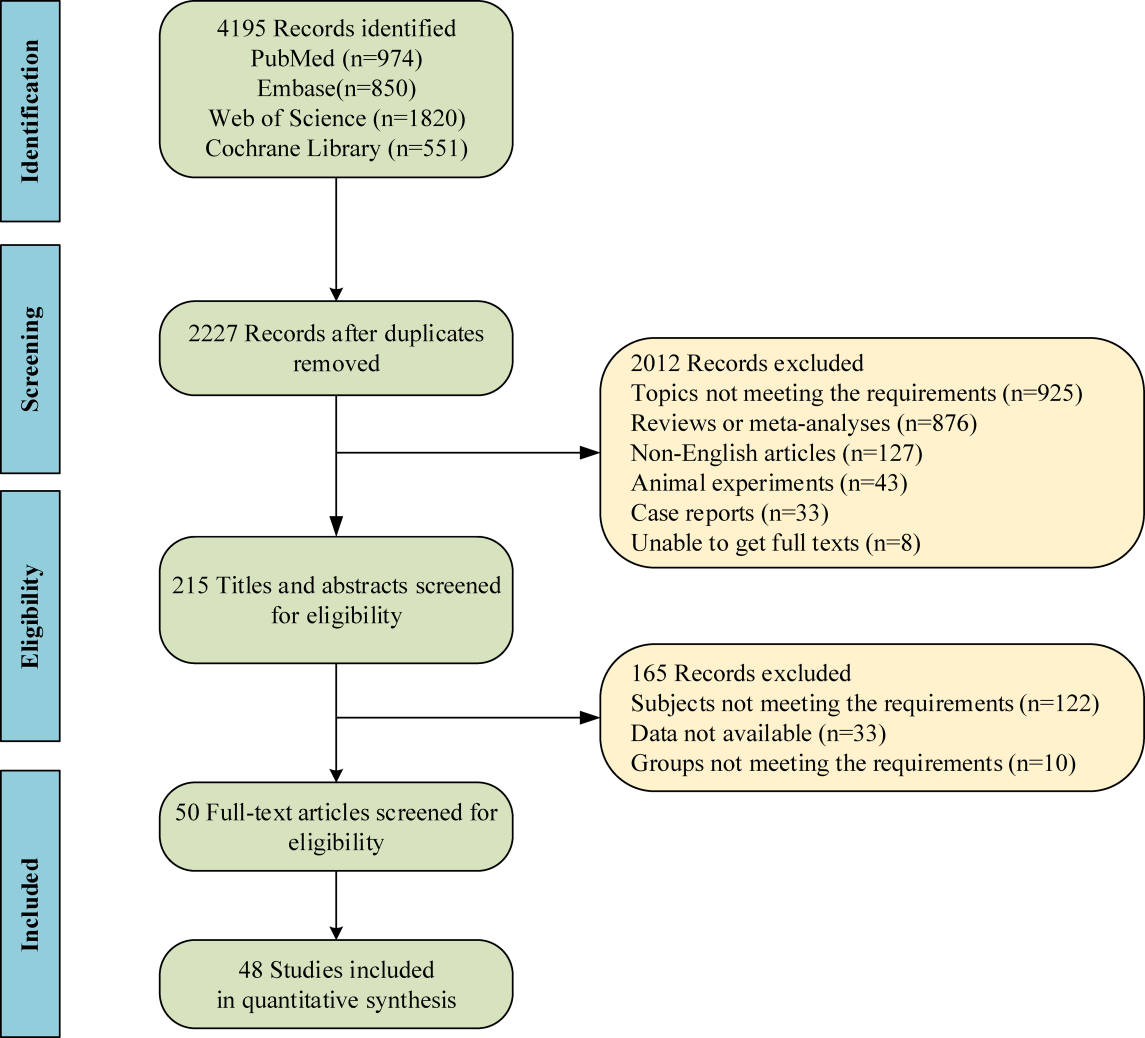
Flow chart of study selection.

A total of 4195 studies were retrieved through database search, and then 1968 duplicates were removed. Based on screening with titles and abstracts, and subsequently with full texts, 50 studies (14–19, 23–33, 40–72) were included in the end, with 48 studies included for quantitative analysis. **Figure 1** shows the screening process of qualified studies. These eligible studies included 10813 patients, with 4122 treated with SLIT, 1852 treated with SCIT, and 4839 treated with non-SLIT or non-SCIT therapy. The year of publication ranged from 1998 to 2023. Six studies (18, 19, 61, 63, 71, 72) made direct comparison between SLIT and SCIT. The features of the included studies are exhibited in **Supplementary Table S1**. Among the included studies, 35 studies were RCTs, of which 8 had low quality and 27 had high quality; 15 were cohort studies, of which 13 had medium quality and 2 had high quality. In RCTs, patients were randomly assigned to the SLIT group or the SCIT group. For cohort studies, the decision for SCIT or SLIT was made based on medical records or parental preference in 6 studies, and 9 studies did not report the grouping basis.

### Direct comparison

#### SLIT versus non-SLIT

##### SSs

SSs were evaluated in 23 studies (15, 17, 24–31, 33, 41–45, 53, 55, 58, 60, 65–67), including 2332 patients in the SLIT group and 2380 in the non-SLIT group. Pooled analysis illustrated that compared with the non-SLIT group, the SLIT group had a significantly lower SS (pooled SMD: -0.99, 95%CI: -1.29, -0.69, I^2 ^= 95.1%, *P* < 0.001) (**Supplementary Figure 1**). Subgroup analysis based on study design, allergen, treatment duration, AIT modality, and AIT protocol found significant differences in SSs between the SLIT and non-SLIT groups when the included studies were RCTs, allergens were HDMs or grass pollen, treatment duration was ≥ 24, 12-23 or < 12 months, AIT modality was drops or tablets, or AIT protocol was continuous, pre- and co-seasonal, or after the GPS (all *P* < 0.05) (**Table 1**).

**Table 1:** Overall results of direct comparison in different outcomes.

Group	Outcome	Number of Studies	Number of patients	SMD/RR (95%CI)	*P*	I^2^
**SLIT vs non-SLIT**	**SSs**					
	Overall	23	2332/2380	-0.99 (-1.29, -0.69)	<0.001	95.1
	Study design					
	RCT	19	2113/2025	-1.07 (-1.41, -0.74)	<0.001	95.5
	Cohort study	4	219/355	-0.65 (-1.34, 0.03)	0.06	92.2
	Allergen					
	HDM	12	698/848	-2.11 (-2.86, -1.36)	<0.001	97.2
	Grass pollen	10	1544/1451	-0.32 (-0.46, -0.19)	<0.001	62.0
	Treatment duration					
	≥24	10	876/995	-1.74 (-2.42, -1.06)	<0.001	97.4
	12-23	6	350/362	-1.36 (-2.19, -0.53)	0.001	94.0
	<12	6	1047/994	-0.31 (-0.39, -0.22)	<0.001	0.0
	AIT modality					
	Drops	17	940/939	-1.60 (-2.16, -1.04)	<0.001	96.2
	Tablets	6	1392/1441	-0.29 (-0.36, -0.22)	<0.001	0.0
	AIT protocol					
	Continuous	15	877/983	-1.82 (-2.44, -1.20)	<0.001	96.8
	Pre-and co-seasonal	6	1036/981	-0.36 (-0.53, -0.19)	<0.001	62.9
	After the GPS	2	391/385	-0.18 (-0.32, -0.04)	0.014	0.0
	**MSs**					
	Overall	17	1980/1902	-0.78 (-1.09, -0.48)	<0.001	94.4
	Study design					
	RCT	14	1798/1731	-0.55 (-0.82, -0.27)	<0.001	92.1
	Cohort study	3	182/171	-1.88 (-3.38, -0.39)	0.014	96.8
	Allergen					
	HDM	7	440/450	-1.64 (-2.73, -0.55)	0.003	97.4
	Grass pollen	9	1450/1371	-0.22 (-0.36, -0.08)	0.002	61.7
	Treatment duration					
	≥24	5	520/510	-1.31 (-2.26, -0.35)	<0.001	97.0
	12-23	5	329/341	-1.41 (-2.68, -0.13)	<0.001	97.0
	<12	6	1072/1022	-0.24 (-0.33, -0.16)	<0.001	0.0
	AIT modality					
	Drops	11	591/464	-1.19 (-1.85, -0.52)	<0.001	95.4
	Tablets	6	1389/1438	-0.19 (-0.28, -0.10)	<0.001	33.0
	AIT protocol					
	Continuous	10	591/554	-1.30 (-2.09, -0.51)	0.001	96.8
	Pre-and co-seasonal	6	1033/978	-0.30 (-0.51, -0.09)	0.004	74.9
	After the GPS	1	300/308	-0.18 (-0.34, -0.02)	0.024	NA
	**SMSs**					
	Overall	6	1067/1005	-0.62 (-0.91, -0.34)	<0.001	86.0
	Allergen					
	HDM	2	260/264	-0.90 (-2.14, 0.35)	0.159	96.2
	Grass pollen	4	807/741	-0.48 (-0.75, -0.21)	<0.001	73.6
	Treatment duration					
	≥24	2	91/83	-1.38 (-1.87, -0.90)	<0.001	45.8
	12-23	1	205/217	-0.28 (-0.47, -0.08)	0.005	NA
	<12	3	771/705	-0.33 (-0.44, -0.23)	<0.001	0.0
	AIT modality					
	Drops	3	223/130	-1.07 (-1.84, -0.30)	0.007	88.6
	Tablets	3	844/875	-0.33 (-0.42, -0.23)	<0.001	0.0
	AIT protocol					
	Continuous	3	279/282	-0.88 (-1.76, 0.01)	0.052	92.8
	Pre-and co-seasonal	4	788/723	-0.44 (-0.72, -0.16)	0.002	77.2
	**New sensitizations**					
	Overall	4	398/408	0.21 (0.04, 1.03)	0.054	92.3
	Study design					
	RCT	2	271/143	0.11 (0.05, 0.22)	<0.001	0.0
	Cohort study	2	127/265	0.44 (0.06, 3.31)	0.427	85.9
	Treatment duration					
	≥24	3	257/331	0.25 (0.03, 1.82)	0.172	92.8
	12-23	1	141/77	0.13 (0.05, 0.33)	<0.001	0.0
	**Development of asthma**					
	Overall	4	663/605	0.43 (0.19, 0.97)	0.042	81.0
	Study design					
	RCT	3	573/524	0.33 (0.09, 1.16)	0.084	87.4
	Cohort study	1	90/81	0.66 (0.36, 1.23)	0.195	NA
	AIT modality					
	Drops	3	265/191	0.30 (0.09, 0.94)	0.039	81.7
	Tablets	1	398/414	0.91 (0.58, 1.41)	0.662	NA
	AIT protocol					
	Continuous	2	220/147	0.20 (0.02, 2.75)	0.23	91.1
	Co-seasonal	1	45/44	0.43 (0.21, 0.89)	0.024	NA
	After the GPS	1	398/414	0.91 (0.58, 1.41)	0.662	NA
	**TRAEs**					
	Overall	22	2965/2624	2.63 (2.44, 2.83)	<0.001	45.2
	Allergen					
	HDM	9	1179/1019	2.99 (2.62, 3.40)	<0.001	34.8
	Grass pollen	13	1786/1605	2.44 (2.23, 2.68)	<0.001	39.5
	Treatment duration					
	≥24	4	503/501	2.46 (2.07, 2.92)	<0.001	38.6
	12-23	6	833/750	3.52 (2.95, 4.19)	<0.001	0.0
	<12	10	1363/1147	2.43 (2.18, 2.70)	<0.001	40.0
	AIT modality					
	drops	10	586/347	2.17 (1.73, 2.73)	<0.001	0.0
	tablets	12	2379/2277	2.70 (2.49, 2.92)	<0.001	65.1
	AIT protocol					
	Continuous	10	1170/1031	2.88 (2.53, 3.27)	<0.001	52.0
	Pre-and co-seasonal	7	1173/1057	2.46 (2.20, 2.76)	<0.001	58.3
	After the GPS	1	398/414	2.67 (2.20, 3.24)	<0.001	NA
**SCIT vs non-SCIT**	**SSs**					
	Overall	5	1279/1223	-2.52 (-3.59, -1.46)	<0.001	95.9
	Study design					
	RCT	1	19/17	-0.54 (-1.21, 0.13)	0.112	NA
	Cohort study	4	1260/1206	-3.06 (-4.30, -1.82)	<0.001	96.2
	Allergen					
	HDM	4	1267/1213	-2.94 (-4.18, -1.70)	<0.001	96.7
	Grass pollen	1	12/10	-0.91 (-1.80, -0.03)	0.044	NA
	Treatment duration					
	≥24	3	162/108	-3.76 (-6.36, -1.16)	0.005	96.3
	12-23	1	1098/1098	-1.88 (-1.98, -1.78)	<0.001	NA
	<12	1	19/17	-0.54 (-1.21, 0.13)	0.112	NA
	AIT protocol					
	Continuous	3	1248/1196	-3.81 (-5.32, -2.30)	<0.001	97.3
	Pre-seasonal	1	12/10	-0.91 (-1.80, -0.03)	0.044	NA
	**MSs**					
	Overall	3	1154/1119	-1.42 (-3.20, 0.36)	0.119	96.9
	Allergen					
	HDM	2	1142/1109	-1.62 (-3.99, 0.75)	0.180	98.0
	Grass pollen	1	12/10	-0.97 (-1.86, -0.08)	0.032	NA
	Treatment duration					
	≥24	2	56/21	-0.60 (-1.15, -0.05)	0.032	5.3
	12-23	1	1098/1098	-2.80 (-2.92, -2.69)	<0.001	NA
	AIT protocol					
	Continuous	2	1142/1109	-1.62 (-3.99, 0.75)	0.180	98.0
	Pre-seasonal	1	12/10	-0.97 (-1.86, -0.08)	0.032	NA
	**SMSs**					
	Overall	2	1110/1108	-2.46 (-5.16, 0.24)	0.074	97.2
	**New sensitizations**					
	Overall	2	66/29	0.62 (0.41, 0.91)	0.016	0.0
	**TRAEs**					
	Overall	2	1108/1108	4.54 (0.30, 68.28)	0.275	88.9
**SLIT vs SCIT**	**SSs**					
	Overall	3	125/207	0.41 (-0.46, 1.28)	0.353	89.6
	Study design					
	RCT	1	34/34	0.00 (-0.48, 0.48)	1.000	NA
	Cohort study	2	91/173	0.62 (-0.46, 1.71)	0.261	84.8
	Treatment duration					
	≥24	1	80/160	1.12 (0.83, 1.40)	<0.001	NA
	12-23	1	34/34	0.00 (-0.48, 0.48)	1.000	NA
	<12	1	11/13	0.00 (-0.80, 0.80)	1.000	NA
	**MSs**					
	Overall	2	114/194	0.82 (-0.88, 2.53)	0.344	97.2

SLIT, sublingual immunotherapy; SCIT, subcutaneous immunotherapy; CI, confidence interval; RCT, randomized controlled trial; AIT, allergen immunotherapy; GPS, grass pollen season; HDM, house dust mite; SSs, symptom scores; MSs, medication scores; SMSs, symptom and medication scores; TRAEs, treatment-related adverse events; NA, not applicable.

##### MSs

MSs were assessed in 17 studies (15, 24–31, 41–44, 53, 55, 60, 65), including 1980 patients in the SLIT group and 1902 in the non-SLIT group. Pooled analysis demonstrated that the SLIT group had a significantly lower MS than the non-SLIT group (pooled SMD: -0.78, 95%CI: -1.09, -0.48, I^2 ^= 94.4%, *P* < 0.001) (**Supplementary Figure 2**). Subgroup analysis based on study design, allergen, treatment duration, AIT modality, and AIT protocol found significant differences in MSs between the SLIT and non-SLIT groups in all subgroups (all *P* < 0.05) (**Table 1**).

##### SMSs

Six studies (25, 27–29, 31, 43) provided data on SMSs, with 1067 patients in the SLIT group and 1005 in the non-SLIT group. Pooled analysis showed that the SLIT group had a significantly lower SMS than the non-SLIT group (pooled SMD: -0.62, 95%CI: -0.91, -0.34, I^2 ^= 86.0%, *P* < 0.001) (**Supplementary Figure 3**). Subgroup analysis based on allergen, treatment duration, AIT modality, and AIT protocol found significant differences in SMSs between the SLIT and non-SLIT groups when the allergen was grass pollen, treatment duration was ≥ 24, 12-23 or < 12 months, AIT modality was drops or tablets, or AIT protocol was pre- or co-seasonal (all *P* < 0.05) (**Table 1**).

##### New sensitizations

Information on new sensitizations was reported by 4 studies (41, 59, 62, 67), with 398 patients in the SLIT group and 408 in the non-SLIT group. Pooled analysis illustrated that the SLIT group had a similar incidence of new sensitizations to the non-SLIT group (pooled RR: 0.21, 95%CI: 0.04, 1.03, I^2 ^= 92.3%, *P* = 0.054) (**Supplementary Figure 4**). Subgroup analysis based on study design and treatment duration showed that the SLIT group had a significantly lower incidence of new sensitizations than the non-SLIT group when the included studies were RCTs or treatment duration was 12-23 months (both *P* < 0.05) (**Table 1**).

##### Development of asthma

Four studies (26, 41, 52, 59) investigated the development of asthma, including 663 patients in the SLIT group and 605 in the non-SLIT group. Pooled analysis exhibited a significantly lower incidence of developing asthma in the SLIT group versus the non-SLIT group (pooled RR: 0.43, 95%CI: 0.19, 0.97, I^2 ^= 81.0%, *P* = 0.042) (**Supplementary Figure 5**). Subgroup analysis based on study design, AIT modality and AIT protocol showed that the SLIT group had a significantly decreased incidence of developing asthma than the non-SLIT group when the AIT modality was drops or AIT protocol was co-seasonal (both *P* < 0.05) (**Table 1**).

##### TRAEs

Twenty-two studies investigated TRAEs (24, 26–29, 31, 32, 42–44, 46, 47, 49, 50, 53, 55, 56, 60, 62, 64, 68, 69), with 2965 patients in the SLIT group and 2624 in the non-SLIT group. According to pooled analysis, the SLIT group had a significantly higher incidence of TRAEs than the non-SLIT group (pooled RR: 2.63, 95%CI: 2.44, 2.83, I^2 ^= 45.2%, *P* < 0.001) (**Supplementary Figure 6**). Subgroup analysis based on allergen, treatment duration, AIT modality, and AIT protocol found significant differences in the incidence of TRAEs between the SLIT and non-SLIT groups in all subgroups (all *P* < 0.05) (**Table 1**).

##### Improvement

The percentage of patients evaluated as “improved” by patients/guardians was significantly higher in the SLIT group (78.8%) than in the placebo group (58.3%) (*P* < 0.0001), which was consistent with the percentage of patients evaluated as general improvement by researchers (67.5% vs 57.4%, *P* = 0.0348) (31). As shown by another study (30), the total effective rate of the SLIT group and the drug only group was 98.08% and 86.00%, respectively (*P* = 0.030). No significant difference was found by de Bot et al. (33) in the overall evaluation of treatment efficacy between the SLIT group and the placebo group (slightly better 33.3% vs 35.8%, much better 21.9% vs 27.5%, no complaints any more 2.9% vs 1.8%). The research of Yonekura et al. (32) illustrated that 33% of patients in the SLIT group improvement of symptoms, compared with 0% in the placebo group.

#### SCIT versus non-SCIT

##### SSs

SSs were evaluated in 5 studies (14, 23, 40, 54, 70), including 1279 patients in the SCIT group and 1223 in the non-SCIT group. Pooled analysis illustrated that compared with the non-SCIT group, the SCIT group had a significantly lower SS (pooled SMD: -2.52, 95%CI: -3.59, -1.46, I^2 ^= 95.9%, *P* < 0.001) (**Supplementary Figure 7**). Subgroup analysis based on study design, allergen, treatment duration, and AIT protocol found significant differences in SSs between the SCIT and non-SCIT groups when the included studies were cohort studies, allergens were HDMs or grass pollen, treatment duration was ≥ 24 or 12-23 months, or AIT protocol was continuous or pre-seasonal (all *P* < 0.05) (**Table 1**).

##### MSs

MSs were assessed in 3 studies (14, 23, 54), including 1154 patients in the SCIT group and 1119 in the non-SCIT group. Pooled analysis demonstrated that the SCIT group had an equivalent MS to the non-SCIT group (pooled SMD: -1.42, 95%CI: -3.20, 0.36, I^2 ^= 96.9%, *P* = 0.119) (**Supplementary Figure 8**). Subgroup analysis based on allergen, treatment duration, and AIT protocol found significant differences in MSs between the SCIT and non-SCIT groups when the allergen was grass pollen, treatment duration was ≥ 24 or 12-23 months, or AIT protocol was continuous or pre-seasonal (all *P* < 0.05) (**Table 1**).

##### SMSs

Two studies (14, 54) provided data on SMSs, with 1110 patients in the SCIT group and 1108 in the non-SCIT group. Pooled analysis showed that the SCIT group had a similar SMS to the non-SCIT group (pooled SMD: -2.46, 95%CI: -5.16, 0.24, I^2^ = 97.2%, *P* = 0.074) (**Table 1**; **Supplementary Figure 9**).

##### New sensitizations

Information on new sensitizations was reported by 2 studies (16, 51), with 66 patients in the SCIT group and 29 in the non-SCIT group. Pooled analysis illustrated that the SCIT group had a significantly lower incidence of new sensitizations than the non-SCIT group (pooled RR: 0.62, 95%CI: 0.41, 0.91, I^2^ = 0.0%, *P* = 0.016) (**Table 1**; **Supplementary Figure 10**).

##### Development of asthma

One study (57) investigated the development of asthma, including 64 patients in the SCIT group and 53 in the non-SCIT group. The SCIT group had a significantly lower incidence of developing asthma than the non-SCIT group (RR: 0.55, 95%CI: 0.33, 0.93, *P* = 0.024).

##### TRAEs

TRAEs were evaluated by 2 studies (48, 61), with 1108 patients in the SCIT group and 1108 in the non-SCIT group. Pooled analysis showed that no significant difference was found in the incidence of TRAEs between the SCIT and non-SCIT groups (pooled RR: 4.54, 95%CI: 0.30, 68.28, I^2^ = 88.9%, *P* = 0.275) (**Table 1**; **Supplementary Figure 11**).

##### Improvement

A study (16) showed that compared with the drug only group alone, the SCIT group also showed a greater improvement in SSs (*P* = 0.0009), with 78.44% of patients feeling “a good deal better” or “slightly better” following SCIT treatment versus 47.06% following drug only therapy.

#### SLIT versus SCIT

##### SSs

**Figure 2:**
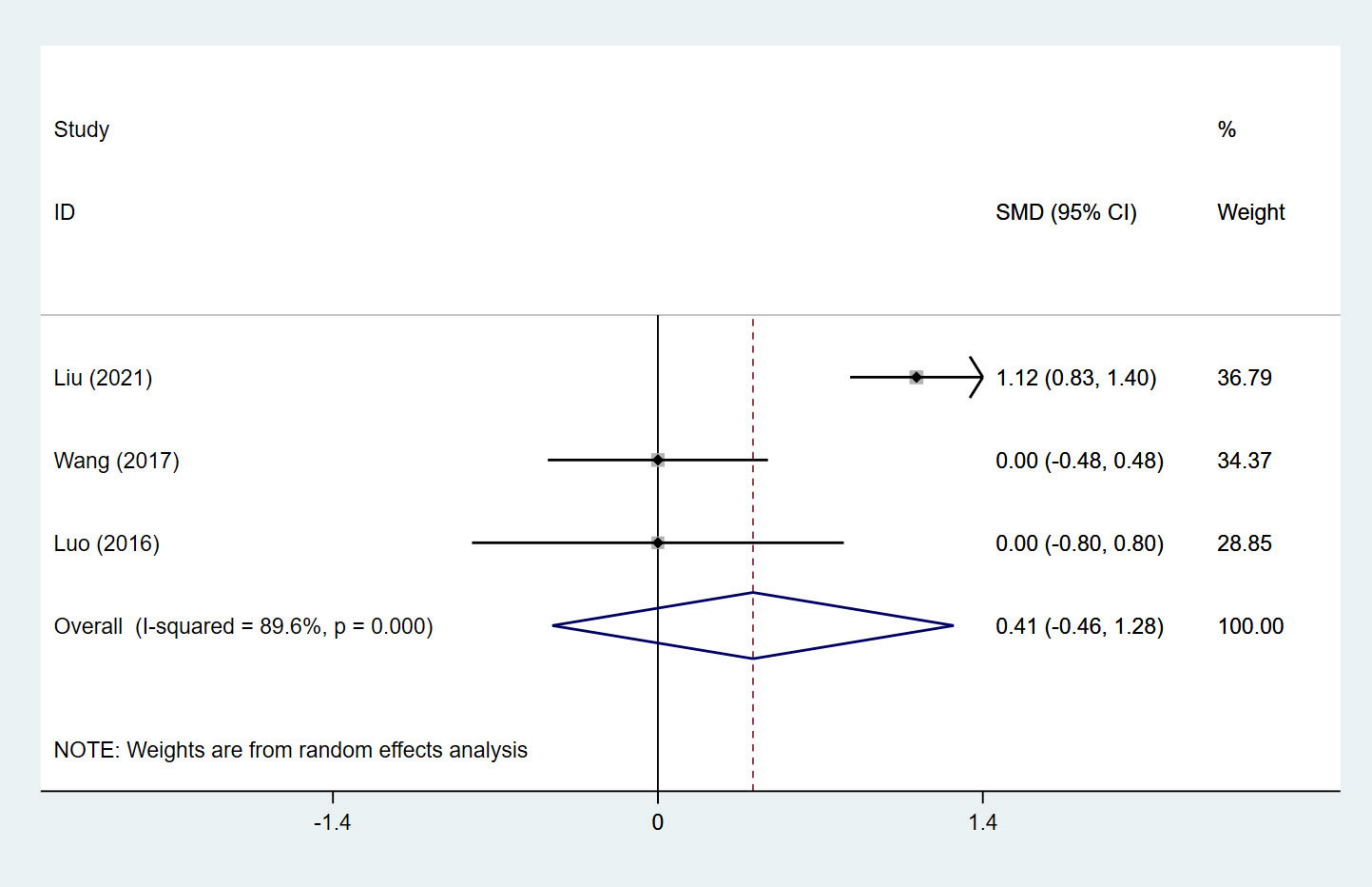
Forest plot for SSs in children receiving SLIT versus SCIT treatment. SLIT, sublingual immunotherapy; SCIT, subcutaneous immunotherapy; SMD, standardized mean differences; CI, confidence interval; SSs, symptom scores.

SSs were evaluated in 3 studies (18, 63, 72), including 125 patients in the SLIT group and 207 in the SCIT group. Pooled analysis illustrated that the SLIT group had a comparable SS to the SCIT group (pooled SMD: 0.41, 95%CI: -0.46, 1.28, I^2^ = 89.6%, *P* = 0.353) (**Figure 2**). Subgroup analysis based on study design and treatment duration found that the SS of the SLIT group was significantly higher than that of the SCIT group when the treatment duration was ≥ 24 months (SMD: 1.12, 95%CI: 0.83, 1.40, *P* < 0.001) (**Table 1**).

##### MSs

**Figure 3:**
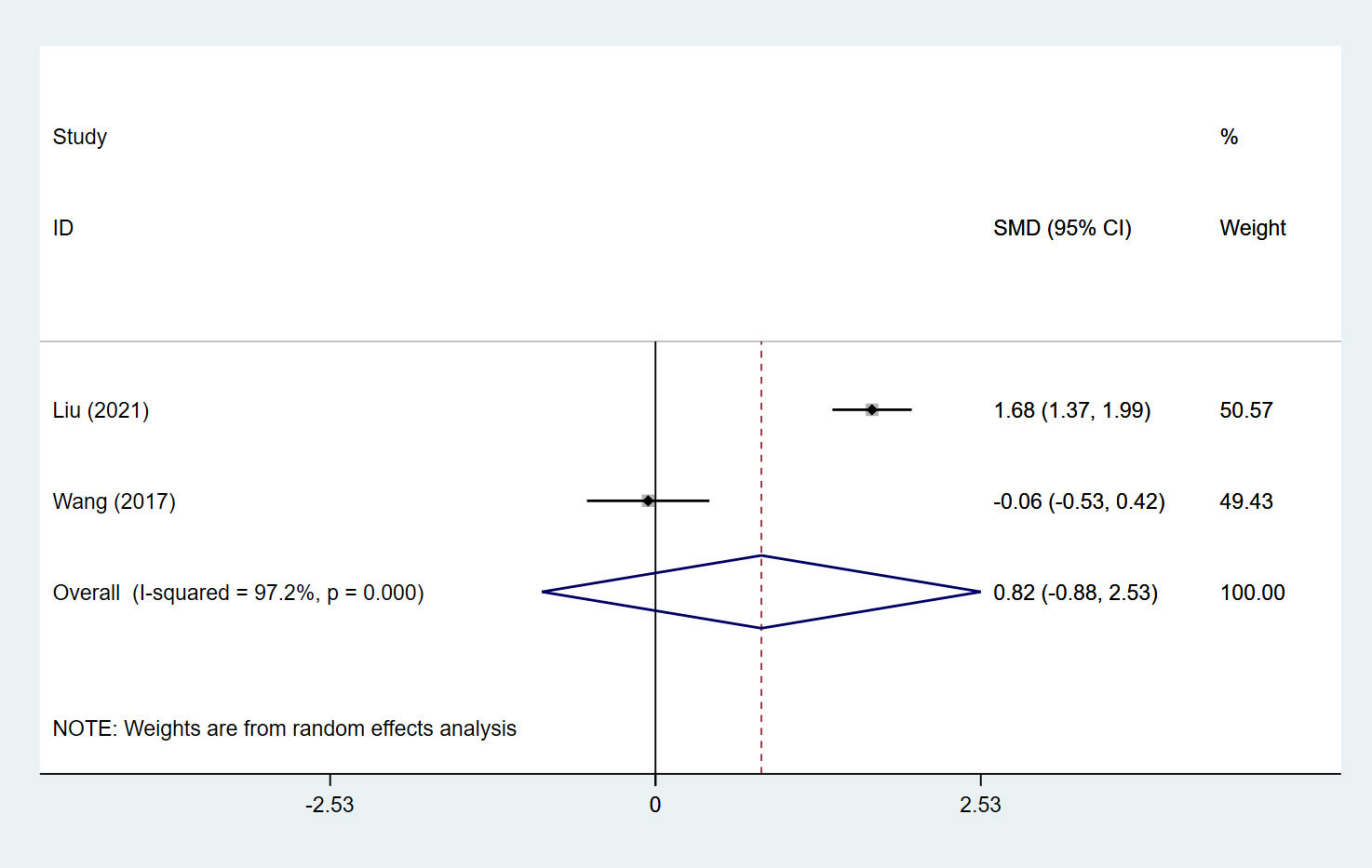
Forest plot for MSs in children receiving SLIT versus SCIT treatment. SLIT, sublingual immunotherapy; SCIT, subcutaneous immunotherapy; SMD, standardized mean differences; CI, confidence interval; MSs, medication scores.

MSs were assessed in 2 studies (18, 72), including 114 patients in the SLIT group and 194 in the SCIT group. Pooled analysis demonstrated no significant difference in MSs between the SLIT and SCIT groups (pooled SMD: 0.82, 95%CI: -0.88, 2.53, I^2^ = 97.2%, *P* = 0.344) (**Table 1**, **Figure 3**).

##### SMSs

One study (72) reported data on SMSs, with 80 patients in the SLIT group and 160 in the SCIT group. The SLIT group had a significantly higher SMS than the SCIT group (SMD: 0.88, 95%CI: 0.60, 1.16, *P* < 0.001).

##### TRAEs

One study (72) assessed TRAEs, with 80 patients in the SLIT group and 160 in the SCIT group. The incidence of TRAEs in the SLIT group was significantly lower than that in the SCIT group (RR: 0.45, 95%CI: 0.23, 0.88, *P* = 0.020).

##### Improvement

As shown by Özdoğru et al. (19), 53.3% of patients in the SCIT group and 61.9% in the SLIT group had self-reported clinical improvement.

### Indirect comparison

#### SLIT versus non-SLIT

Compared with the non-SLIT group, the SLIT group had a significantly lower incidence of new sensitizations (pooled RR: 0.21, 95%CrI: 0.05, 0.83), and exhibited a significantly higher incidence of TRAEs (pooled RR: 3.40, 95%CrI: 2.10, 5.50) (**Table 2**).

**Table 2:** Respective overall results of direct and indirect comparisons in different outcomes [SMD/RR (95%CI)].

	SLIT vs non-SLIT		SCIT vs non-SCIT		SLIT vs SCIT	
	Direct	Indirect	Direct	Indirect	Direct	Indirect
**SSs**	-0.99 (-1.29, -0.69)	-2.10 (-5.80, 1.60)	-2.52 (-3.59, -1.46)	-1.90 (-5.50, 1.60)	0.41 (-0.46, 1.28)	1.20 (-1.70, 4.10)
**MSs**	-0.78 (-1.09, -0.48)	-0.69 (-2.60, 1.20)	-1.42 (-3.20, 0.36)	-1.70 (-3.70, 0.39)	0.82 (-0.88, 2.53)	0.57 (-1.20, 2.30)
**SMSs**	-0.62 (-0.91, -0.34)	-1.20 (-3.30, 0.79)	-2.46 (-5.16, 0.24)	-1.70 (-3.90, 0.45)	0.88 (0.60, 1.16)	1.80 (-0.005, 3.60)
**New sensitizations**	0.21 (0.04, 1.03)	0.21 (0.05, 0.83)	0.62 (0.41, 0.91)	0.62 (0.09, 4.38)	–	0.34 (0.03, 3.58)
**Development of asthma**	0.43 (0.19, 0.97)	0.37 (0.06, 1.86)	0.55 (0.33, 0.93)	0.54 (0.02, 15.27)	–	0.68 (0.01, 26.33)
**TRAEs**	2.63 (2.44, 2.83)	3.40 (2.10, 5.50)	4.54 (0.30, 68.28)	7.20 (3.60, 14.00)	0.45 (0.23, 0.88)	0.17 (0.11, 0.26)

SLIT, sublingual immunotherapy; SCIT, subcutaneous immunotherapy; SMD, standardized mean differences; RR, relative risk; CI, confidence interval; SSs, symptom scores; MSs, medication scores; SMSs, symptom and medication scores; TRAEs, treatment-related adverse events.

#### SCIT versus non-SCIT

The incidence of TRAEs in the SCIT group was significantly higher than that in the non-SCIT group (pooled RR: 7.20, 95%CrI: 3.60, 14.00) (**Table 2**).

#### SLIT versus SCIT

In contrast to the SCIT group, the SLIT group illustrated a significantly lower incidence of TRAEs (pooled RR: 0.17, 95%CrI: 0.11, 0.26) (**Table 2**).

### Source of heterogeneity and sensitivity analysis

The result of meta-regression showed that treatment duration was a source of heterogeneity in assessing SLIT versus non-SLIT for SMSs (*P*=0.006) (**Supplementary Table S2**). According to sensitivity analysis, one-study deletion did not have a significant impact on the pooled results, suggesting that the findings of this meta-analysis were stable and robust.

## Discussion

To the best of our knowledge, this systematic review and meta-analysis is the first to compare SCIT and SLIT for SSs, MSs, SMSs, new sensitizations, development of asthma, improvement, and TRAEs in children with AR through both direct and indirect comparisons. It was found that SCIT and SLIT may have comparable effects on SSs, MSs, SMSs, new sensitizations, and development of asthma. For safety, patients undergoing SLIT may exhibit a significantly lower incidence of TRAEs than those undergoing SCIT. These findings indicated that considering both efficacy and safety, SLIT might be superior to SCIT in the treatment of pediatric AR. Clinicians may make AIT choices for AR in children based on the above findings.

Several studies have been conducted to systematically evaluate the efficacy of SCIT versus SLIT among patients with AR. The meta-analysis of Dretzke et al. (20) on the general population with seasonal AR obtained inconclusive results for the superiority of SCIT or SLIT over the other treatment in terms of SSs, MSs, SMSs, and quality-of-life scores. Another meta-analysis demonstrated that patients with seasonal AR to grass pollen receiving SCIT had better control of symptoms and less use of medications than those receiving SLIT (73). Tie et al. (22) compared SCIT and SLIT in adult AR patients via a meta-analysis, and reported similar effects of the two immunotherapies regarding SSs, MSs and SMSs. The current study paid attention to children with AR, and made direct and indirect comparisons between SCIT and SLIT in terms of SSs, MSs, SMSs, new sensitizations, development of asthma, improvement, and TRAEs. With head-to-head comparison, pooled analysis showed that compared with non-SLIT treatment, SLIT was more effective concerning SSs, MSs, SMSs, development of asthma, and TRAEs; AR patients receiving SCIT had lower SSs and reduced new sensitizations than those receiving non-SCIT treatment, suggesting that SCIT and SLIT were superior to non-SCIT and non-SLIT therapy for pediatric AR. In seasonal AR, SLIT was shown to relieve symptoms by 30 to 40% and prescription use (74). A previous review found that AR patients treated with SLIT had reduced symptoms and need for medications than those receiving placebo, and indicated that SLIT causes notable changes in allergen-specific IgG and IgG_4_ antibodies, which is consistent with clinical responses regarding SSs and MSs (75). The efficacy of SCIT among individuals with AR was identified by prior meta-analyses (76, 77). SCIT was reported to be effective in terms of SSs compared with the placebo control in seasonal AR (78). Besides, Alvaro-Lozano et al. (79) demonstrated that SCIT could lower the occurrence of new allergen sensitization in asthmatic children. SCIT and SLIT may be preferred in the treatment of pediatric AR to non-SCIT and non-SLIT treatment, while close attention should be paid to TRAEs during SCIT or SLIT.

Of the 50 included studies (14–19, 23–33, 40–72), merely 6 studies (18, 19, 61, 63, 71, 72) directly compared SLIT and SCIT, no studies directly compared the effects of SLIT and SCIT on new sensitizations and development of asthma, and only one study (72) provided the direct comparison of SLIT and SCIT for SMSs and TRAEs despite significant differences between these two treatments. Thus, indirect comparison was conducted to further assess the efficacy and safety of SLIT versus SCIT. According to direct and indirect evidence, regarding efficacy, SLIT and SCIT may display the equivalent effects on SSs, MSs, SMSs, new sensitizations, and development of asthma. Since limited studies qualified for the direct comparison of SLIT and SCIT in pediatric AR, more well-designed studies are required to directly compare the two routes of administration in the future, which may strengthen the results of this meta-analysis. Besides, two included studies provided qualitative evidence for SCIT versus SLIT in terms of SSs and MSs: Proctor et al. (71) showed that after 3 years of intervention, patients with pollen SCIT, pollen SLIT, or HDM SLIT improved their VAS scores by about 50%, with no significant difference among the three groups. The study by Yukselen et al. (61) reported similar effects of SCIT and SLIT in the reduction of rhinitis symptoms (*P*=0.28) or MSs related to rhinitis (*P*=0.18) and asthma (*P*=0.31). Compared with the SLIT group, only asthma symptoms were significantly reduced in the SCIT group (*P*=0.01). Since the inconsistent definition of improvement in the included studies, and the difficulty in pooled analysis, qualitative descriptions was made for this outcome. Relevant data under a unified definition are needed to quantitatively explore the role of these two treatments in improvement. A prior review illustrated that both SLIT and SCIT played an effective role in reducing symptoms and need for additional medication for AR patients (11). Nelson et al. (80) similarly reported comparable decreases in allergic symptoms and rescue medication intake after using SLIT tablets and SCIT for grass pollen allergies. Concerning the possible similar effectiveness of SLIT and SCIT, these two routes of therapy may function under similar mechanisms. For example, SLIT and SCIT may cause similar generation of IgG antibodies, activity of FOXP3^+^ CD25^+^ Treg cells, and allergen-specific tolerance in pediatric AR (76). Specific underlying mechanisms of SLIT and SCIT in children with AR are worth further exploration. Restoring immunological tolerance to allergens is the main objective of AIT’s mechanism of action, which has been demonstrated to entail many immunologic pathways and the interaction of the innate and adaptive immune responses (79, 81). Of note, concerning safety, a lower incidence of TRAEs were found after SLIT versus SCIT in the current analysis, indicating that SLIT appears to have a better safety profile than SCIT. Likewise, Ji et al. (82) reported that patients undergoing SLIT had fewer adverse reactions. Another study showed that adverse responses were more common with SCIT compared with SLIT (83). SCIT can cause serious adverse events and even anaphylactic shock, while SLIT can be safely self-administered, and local adverse reactions (primarily limited to oral discomfort) caused by SLIT are often mild and subside without treatment (11, 82, 84, 85).

Apart from efficacy and safety, the convenience and cost-effectiveness of these two treatment methods also need to be considered in clinical treatment choices. Subcutaneous injection (SCIT) is regarded as a time-consuming and invasive therapeutic method (86). As a self-administered alternative to SCIT, SLIT offers the advantages of AIT without the expense and inconvenience of frequent office visits or the discomfort of injections (87, 88). Meadows et al. (89) showed that both SCIT and SLIT may be cost-effective compared with symptomatic therapy after about 6 years (threshold of £20000-30000 per quality-adjusted life-year) in AR. As exhibited by previous studies, SCIT was more cost-effective than SLIT in children and adults with AR, with slightly higher patient adherence and lower pharmacological expenditures (90–92). However, Hardin et al. (93) demonstrated that in contrast to SCIT, SLIT is financially beneficial, and should be seen as an economically conscious choice for patients with >40% treatment compliance. SLIT provides the benefit over SCIT in that it does not require injections (94). In young children, injections are less acceptable (95). Due to inconsistent evidence, future investigations are necessary to compare the cost-effectiveness of SLIT and SCIT, which may depend on the local health system.

This meta-analysis comprehensively compared the efficacy and safety of SLIT and SCIT in children with AR using the direct and indirect evidence. In clinical practice, SLIT and SCIT may both be applied to manage symptoms, medication use and development of new sensitizations and asthma related to AR, while SLIT may be preferred as regards adverse events. Convenience and cost-effectiveness as well as relevant clinical experience, patient preference and adherence should also be taken into account by clinicians in the treatment of children with AR. Some limitations should be acknowledged. First, the heterogeneity of the results was high. The meta-regression found that treatment duration was a source of heterogeneity. There may be other sources of heterogeneity, such as the drugs used, dosage administered, etc., which necessitates future research to further assess the source of heterogeneity. Second, the diagnostic criteria for AR in the included studies were not entirely consistent, while most studies were based on skin prick tests or immunoglobulin E (IgE) levels for diagnosis. Due to almost not exactly the same diagnostic methods and limited data, the subgroup analysis cannot be achieved. Studies in the future should standardize the diagnostic criteria for AR to improve equivalence between patients for pooled analysis. Besides, the demographic data of the included patients were inadequate for assessing the role of demographic factors on the outcome of each treatment, indicating that future investigations should improve the reporting of demographic data to facilitate relevant assessment. Third, the lack of studies on some outcomes (e.g. new sensitizations, development of asthma) may have affected the stability of the results. Finally, there were a relatively small number of studies on direct comparison between SLIT and SCIT, and more head-to-head studies are needed in the future to enhance the reliability of the findings.

## Conclusion

SCIT and SLIT may have similar effects on SSs, MSs, SMSs, new sensitizations, and development of asthma, while SLIT may be superior to SCIT in terms of TRAEs in children with AR. Considering both efficacy and safety, SLIT might be a more favorable AIT than SCIT in the treatment of pediatric AR. Future large-scale studies for head-to-head comparisons of SCIT and SLIT are warranted to verify our findings.

## Data availability statement

The original contributions presented in the study are included in the article/**Supplementary Material**. Further inquiries can be directed to the corresponding author.

## Author contributions

JY: Conceptualization, Data curation, Formal analysis, Methodology, Supervision, Writing – original draft, Writing – review & editing. SL: Conceptualization, Data curation, Formal analysis, Writing – review & editing.

## Supplementary material

Supplementary Figure 1 Forest plot for SSs in children receiving SLIT versus non-SLIT treatment. SLIT, sublingual immunotherapy; SMD, standardized mean differences; CI, confidence interval; SSs, symptom scores.

Supplementary Figure 2 Forest plot for MSs in children receiving SLIT versus non-SLIT treatment. SLIT, sublingual immunotherapy; SMD, standardized mean differences; CI, confidence interval; MSs, medication scores.

Supplementary Figure 3 Forest plot for SMSs in children receiving SLIT versus non-SLIT treatment. SLIT, sublingual immunotherapy; SMD, standardized mean differences; CI, confidence interval; SMSs, symptom and medication scores.

Supplementary Figure 4 Forest plot for new sensitizations in children receiving SLIT versus non-SLIT treatment. SLIT, sublingual immunotherapy; SMD, standardized mean differences; CI, confidence interval.

Supplementary Figure 5 Forest plot for development of asthma in children receiving SLIT versus non-SLIT treatment. SLIT, sublingual immunotherapy; RR, relative risk; CI, confidence interval.

Supplementary Figure 6 Forest plot for TRAEs in children receiving SLIT versus non-SLIT treatment. SLIT, sublingual immunotherapy; RR, relative risk; CI, confidence interval; TRAEs, treatment-related adverse events.

Supplementary Figure 7 Forest plot for SSs in children receiving SCIT versus non-SCIT treatment. SCIT, subcutaneous immunotherapy; SMD, standardized mean differences; CI, confidence interval; SSs, symptom scores.

Supplementary Figure 8 Forest plot for MSs in children receiving SCIT versus non-SCIT treatment. SCIT, subcutaneous immunotherapy; SMD, standardized mean differences; CI, confidence interval; MSs, medication scores.

Supplementary Figure 9 Forest plot for SMSs in children receiving SCIT versus non-SCIT treatment. SCIT, subcutaneous immunotherapy; SMD, standardized mean differences; CI, confidence interval; SMSs, symptom and medication scores.

Supplementary Figure 10 Forest plot for new sensitizations in children receiving SCIT versus non-SCIT treatment. SCIT, subcutaneous immunotherapy; RR, relative risk; CI, confidence interval.

Supplementary Figure 11 Forest plot for TRAEs in children receiving SCIT versus non-SCIT treatment. SCIT, subcutaneous immunotherapy; RR, relative risk; CI, confidence interval; TRAEs, treatment-related adverse events.
