# Supplementary figures and images for: Efficacy and safety of sublingual versus subcutaneous immunotherapy in children with allergic rhinitis: a systematic review and meta-analysis
Source: Front Immunol. 2023 Dec 15;14:1274241. doi: 10.3389/fimmu.2023.1274241 (PMC10757840; doi:10.3389/fimmu.2023.1274241)

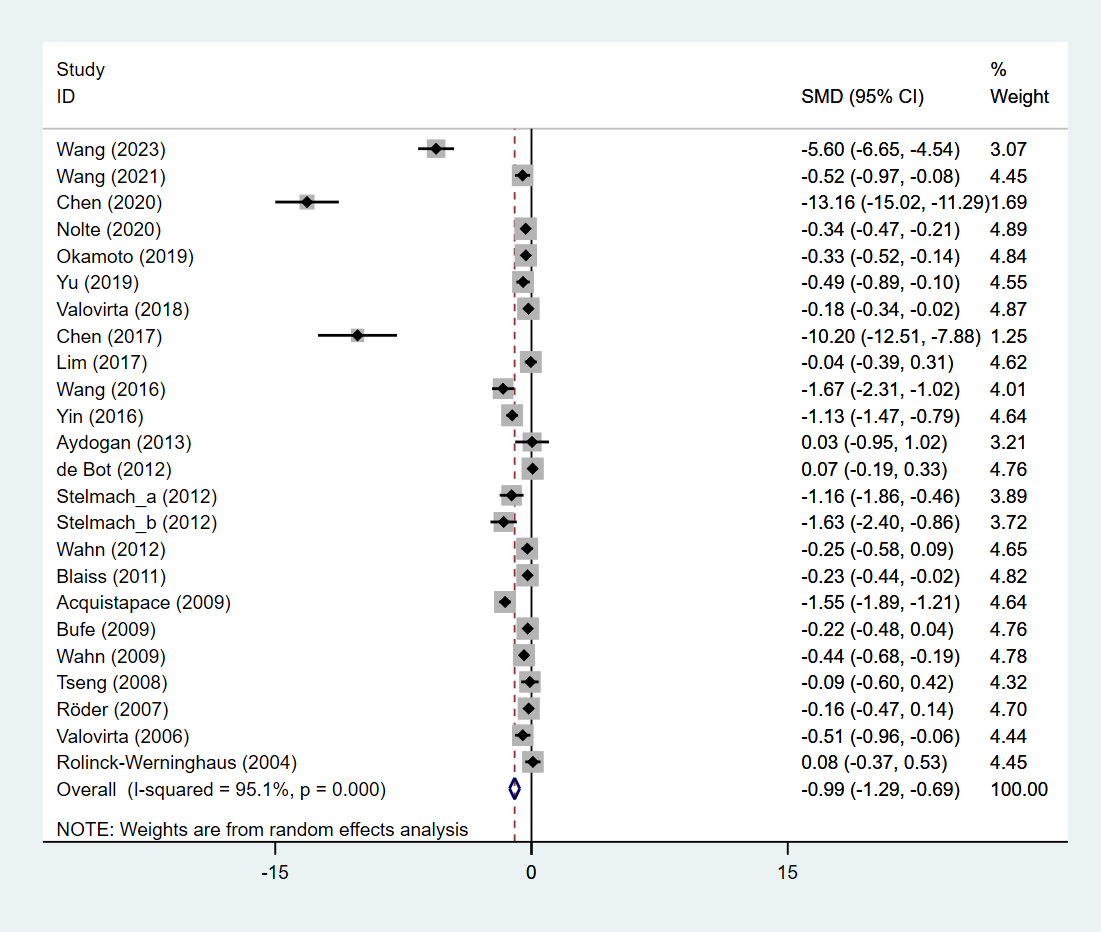

Supplement: Supplementary Figure 1 — Forest plot for SSs in children receiving SLIT versus non-SLIT treatment. SLIT, sublingual immunotherapy; SMD, standardized mean differences; CI, confidence interval; SSs, symptom scores. [file Image_1.tif]

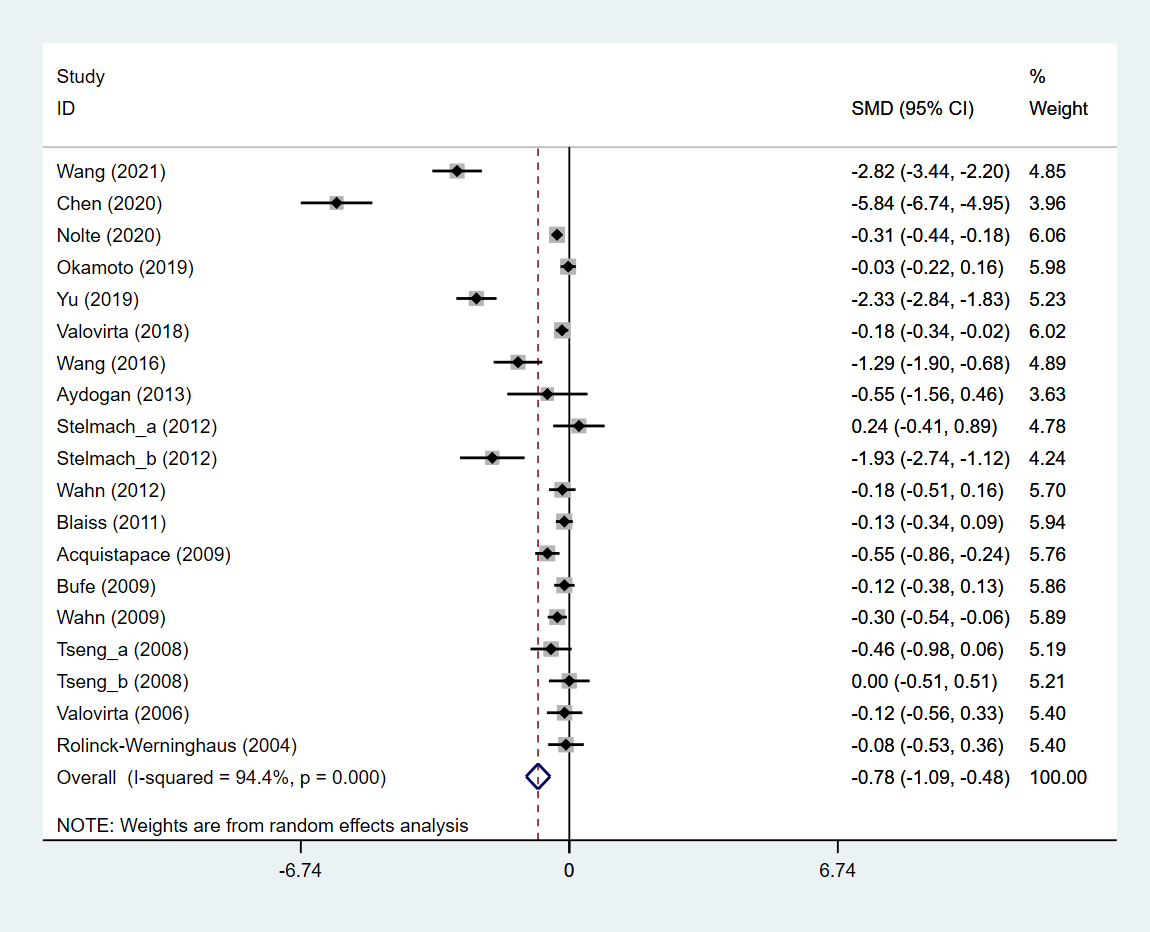

Supplement: Supplementary Figure 2 — Forest plot for MSs in children receiving SLIT versus non-SLIT treatment. SLIT, sublingual immunotherapy; SMD, standardized mean differences; CI, confidence interval; MSs, medication scores. [file Image_2.tif]

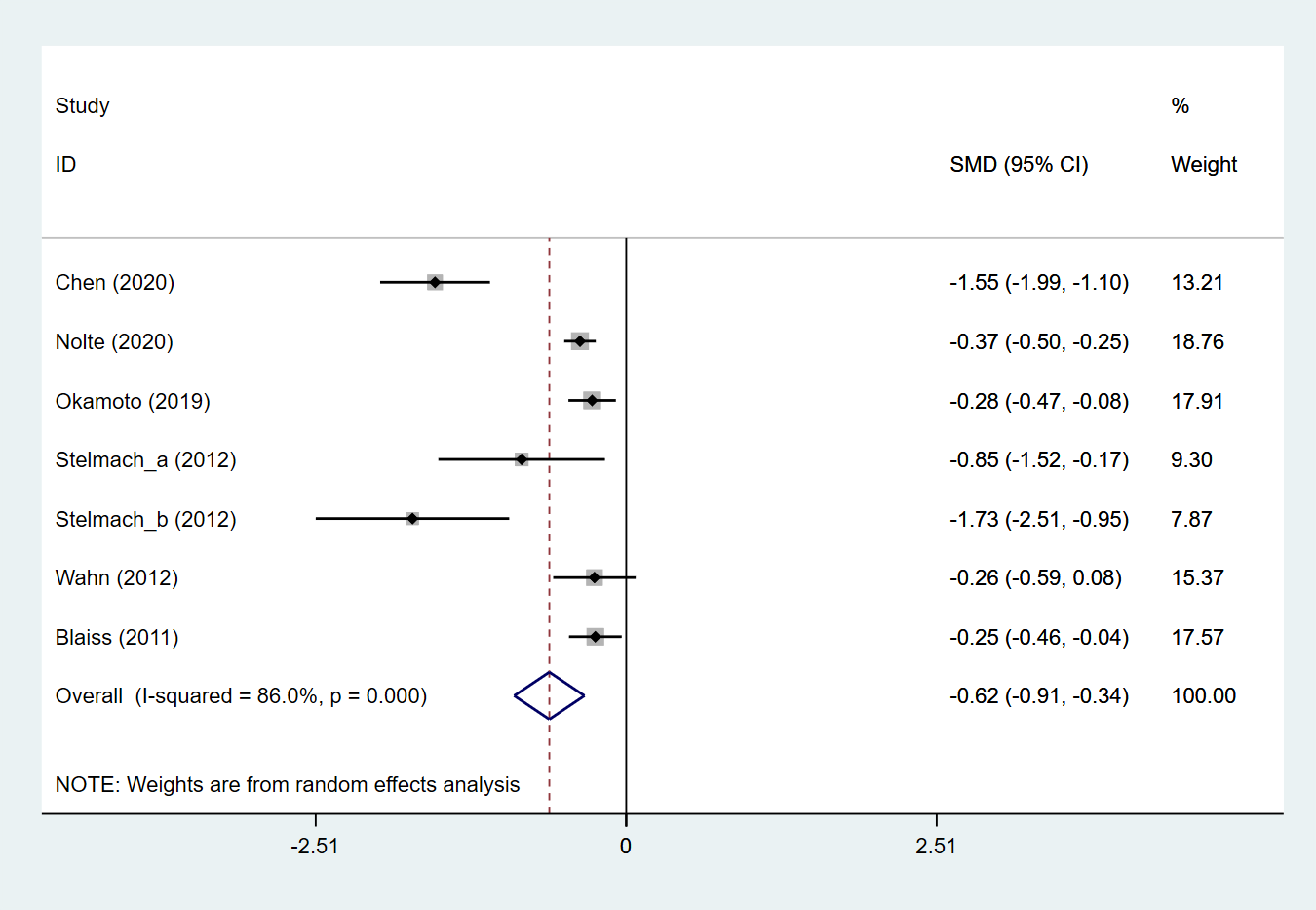

Supplement: Supplementary Figure 3 — Forest plot for SMSs in children receiving SLIT versus non-SLIT treatment. SLIT, sublingual immunotherapy; SMD, standardized mean differences; CI, confidence interval; SMSs, symptom and medication scores. [file Image_3.tif]

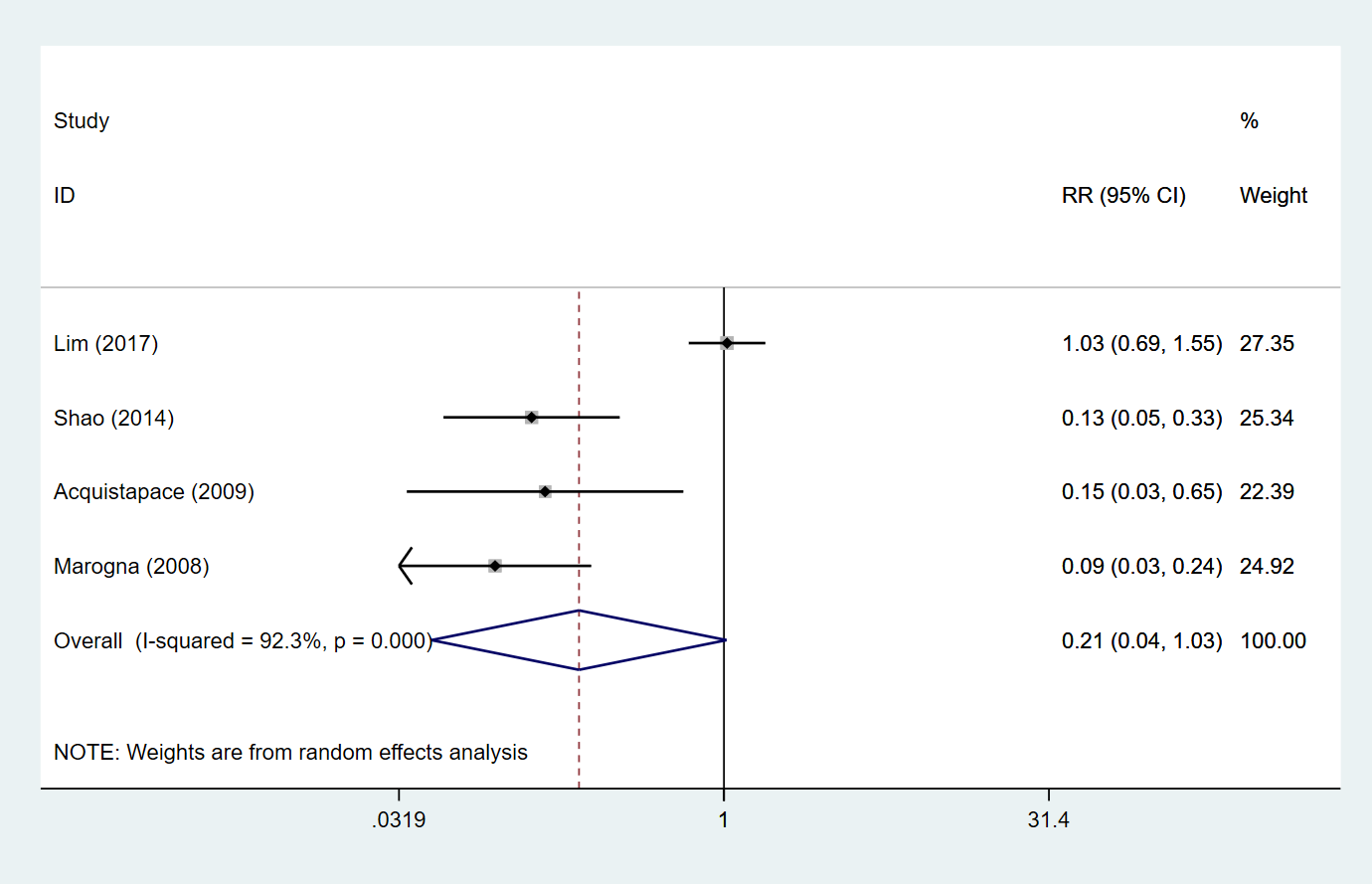

Supplement: Supplementary Figure 4 — Forest plot for new sensitizations in children receiving SLIT versus non-SLIT treatment. SLIT, sublingual immunotherapy; SMD, standardized mean differences; CI, confidence interval. [file Image_4.tif]

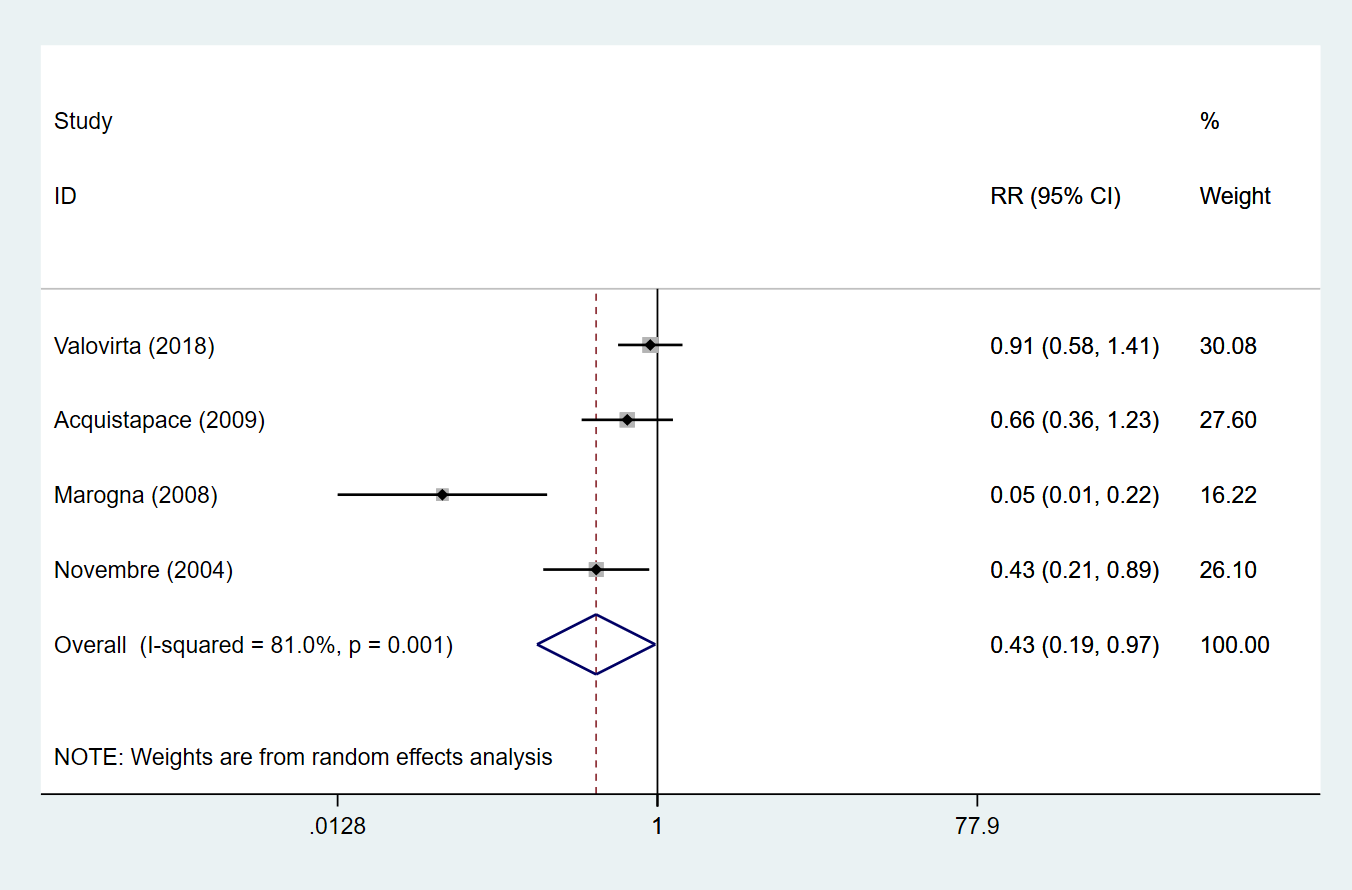

Supplement: Supplementary Figure 5 — Forest plot for development of asthma in children receiving SLIT versus non-SLIT treatment. SLIT, sublingual immunotherapy; RR, relative risk; CI, confidence interval. [file Image_5.tif]

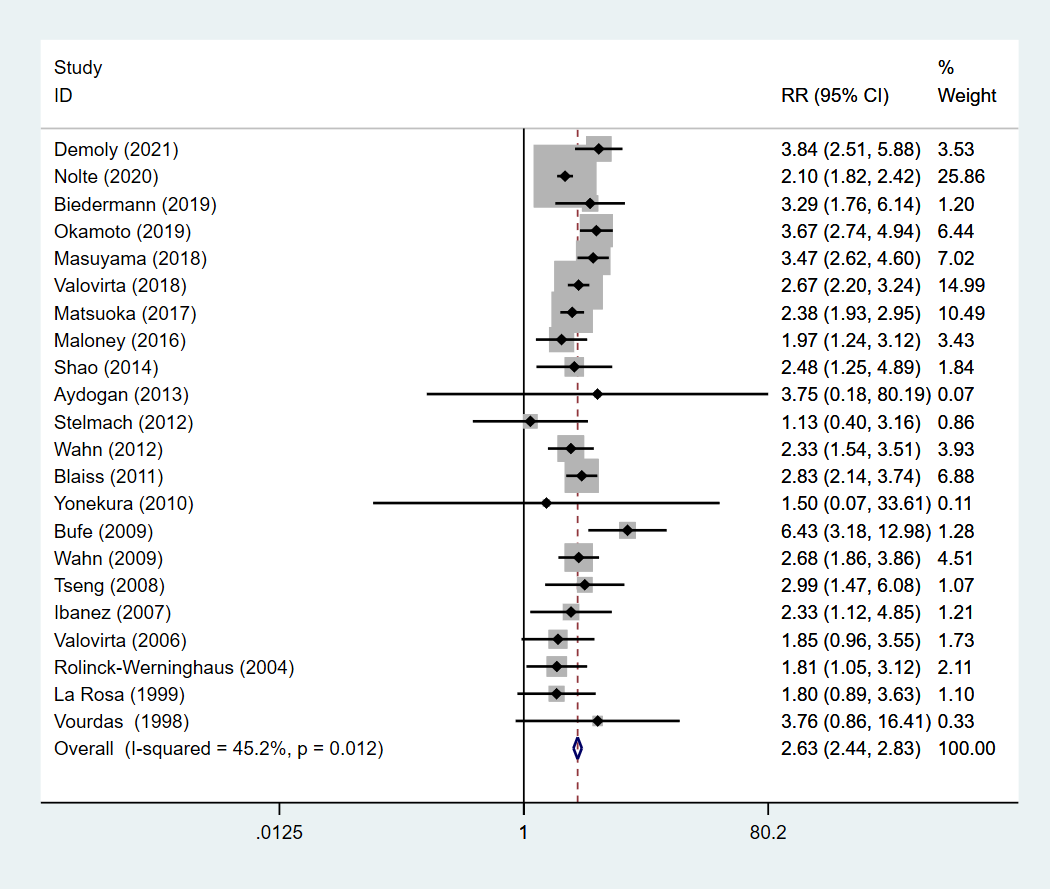

Supplement: Supplementary Figure 6 — Forest plot for TRAEs in children receiving SLIT versus non-SLIT treatment. SLIT, sublingual immunotherapy; RR, relative risk; CI, confidence interval; TRAEs, treatment-related adverse events. [file Image_6.tif]

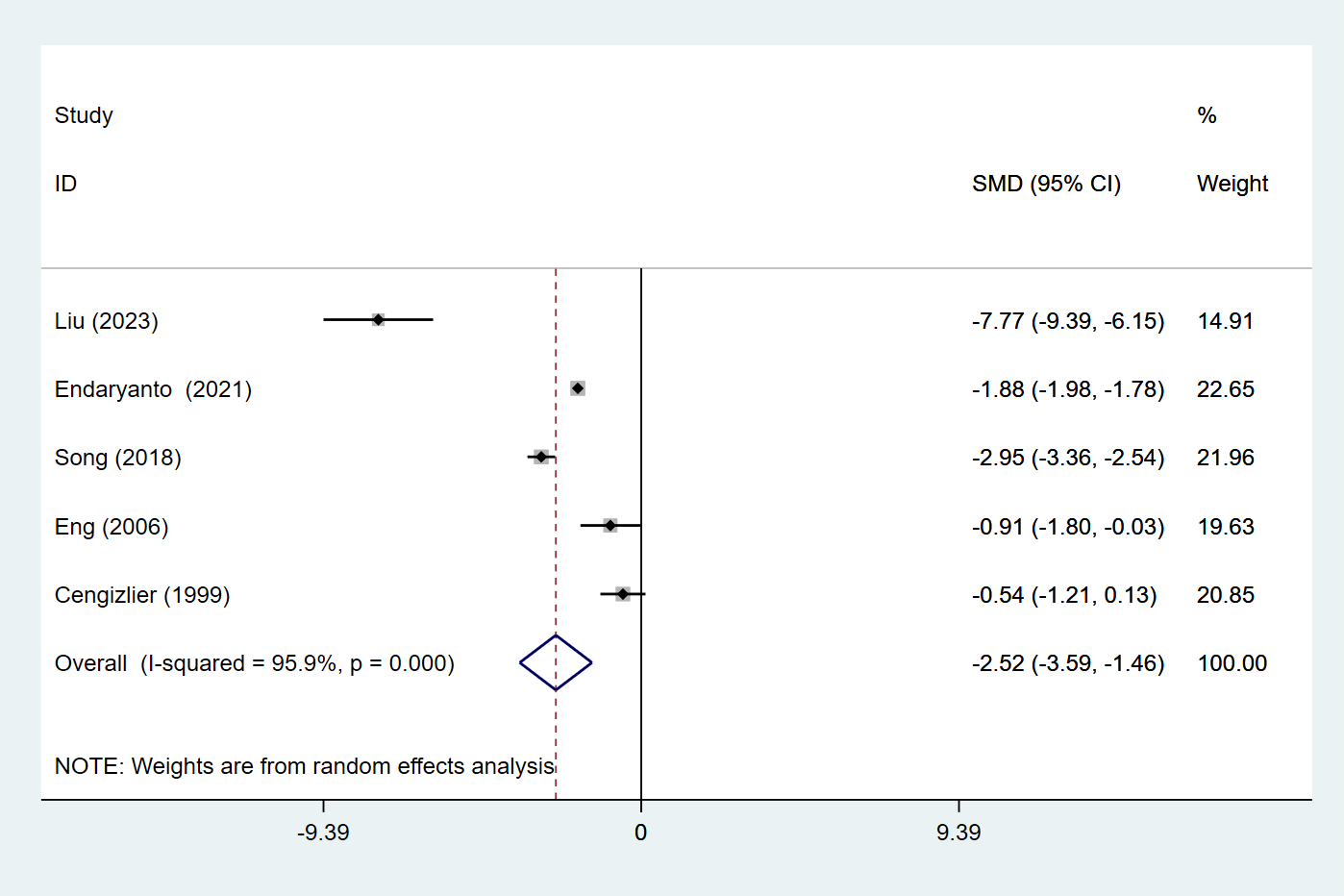

Supplement: Supplementary Figure 7 — Forest plot for SSs in children receiving SCIT versus non-SCIT treatment. SCIT, subcutaneous immunotherapy; SMD, standardized mean differences; CI, confidence interval; SSs, symptom scores. [file Image_7.tif]

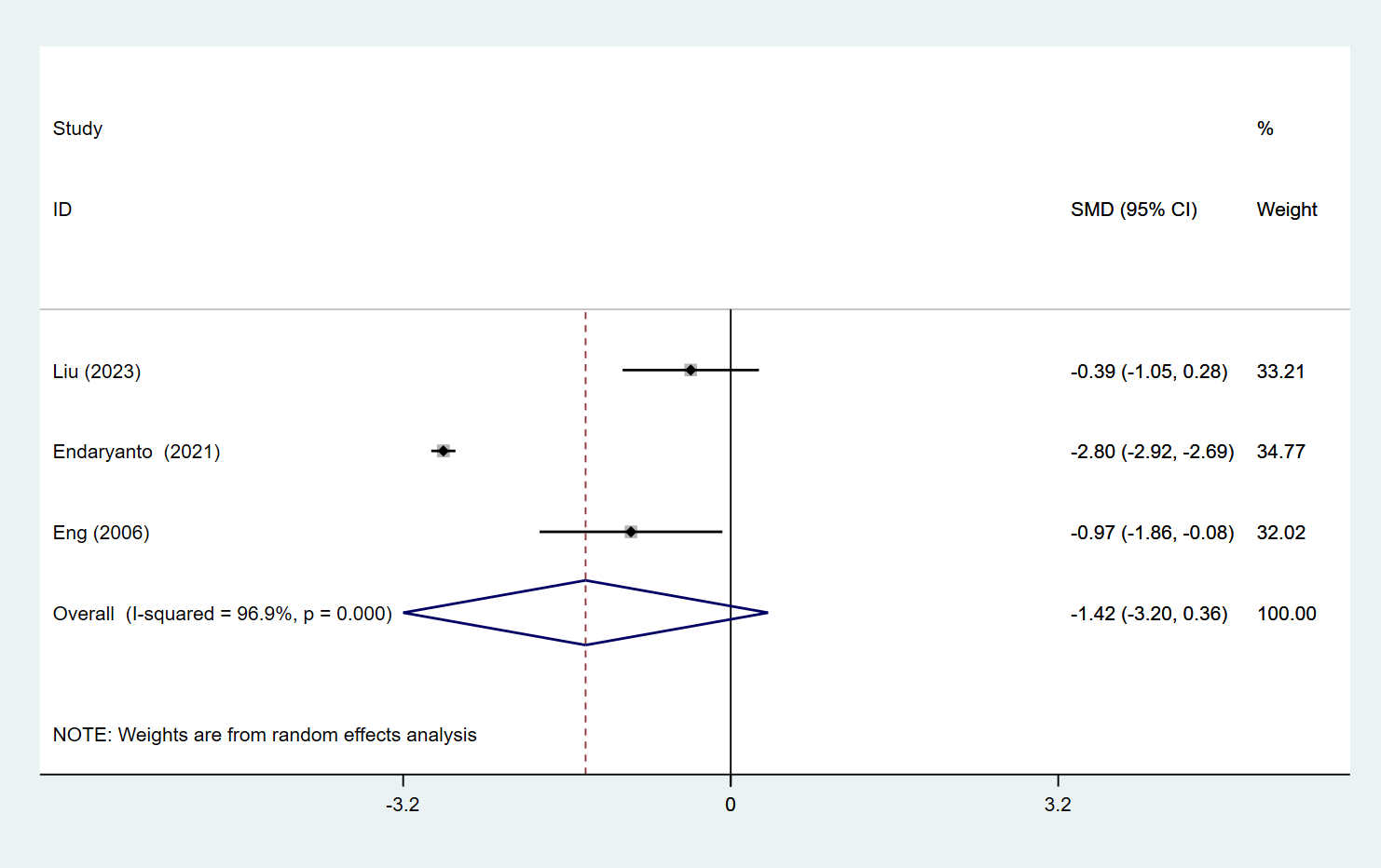

Supplement: Supplementary Figure 8 — Forest plot for MSs in children receiving SCIT versus non-SCIT treatment. SCIT, subcutaneous immunotherapy; SMD, standardized mean differences; CI, confidence interval; MSs, medication scores. [file Image_8.tif]

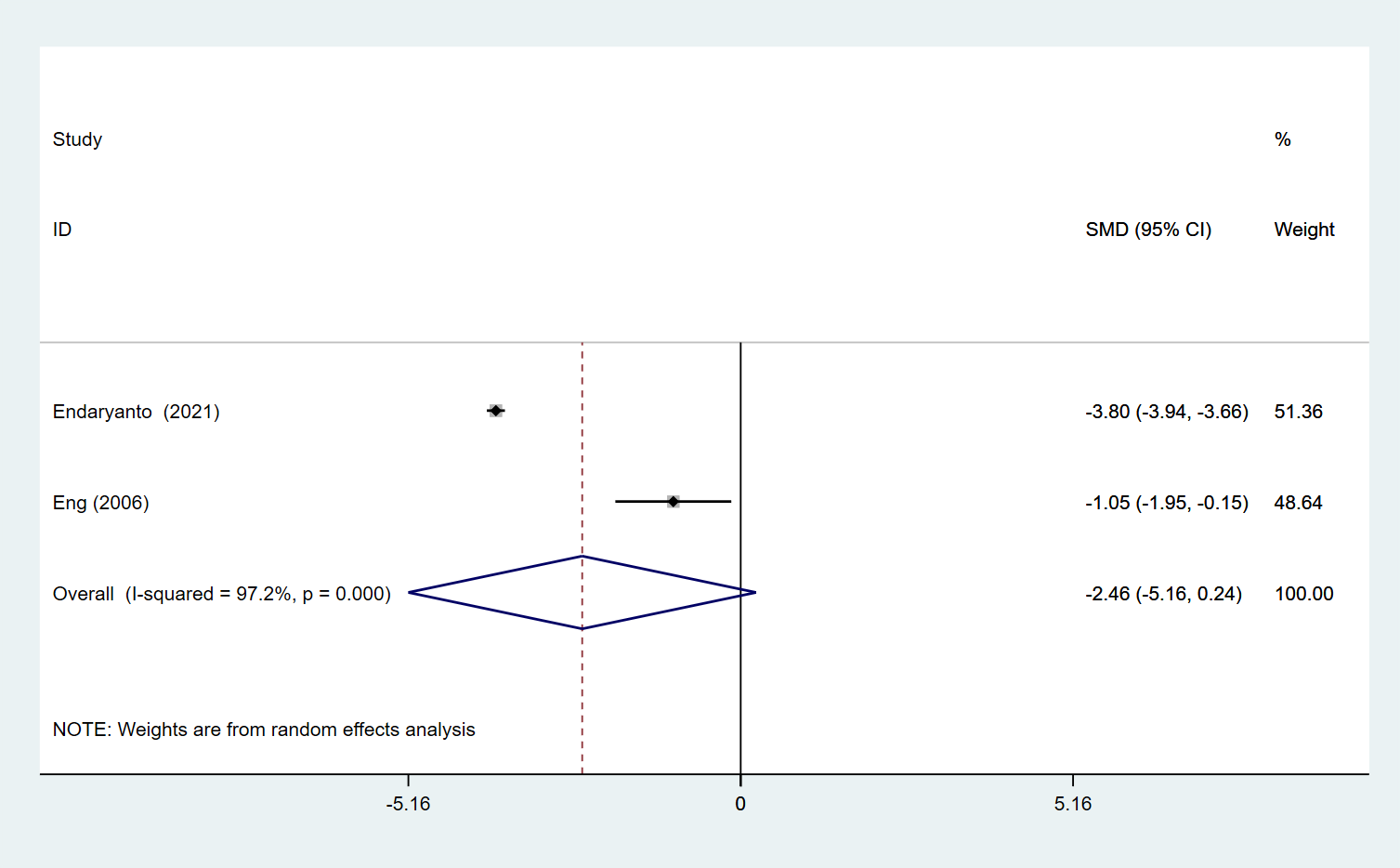

Supplement: Supplementary Figure 9 — Forest plot for SMSs in children receiving SCIT versus non-SCIT treatment. SCIT, subcutaneous immunotherapy; SMD, standardized mean differences; CI, confidence interval; SMSs, symptom and medication scores. [file Image_9.tif]

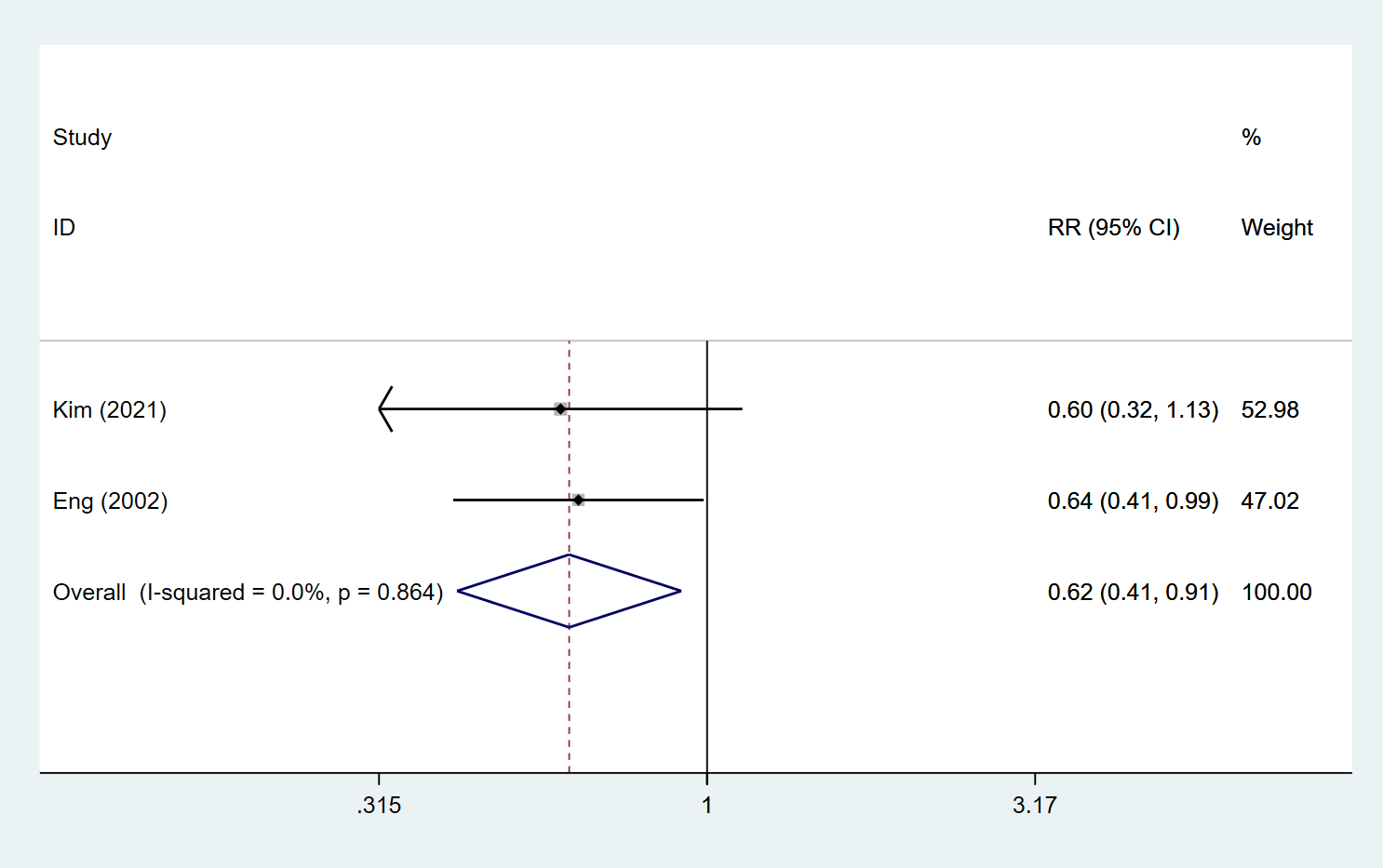

Supplement: Supplementary Figure 10 — Forest plot for new sensitizations in children receiving SCIT versus non-SCIT treatment. SCIT, subcutaneous immunotherapy; RR, relative risk; CI, confidence interval. [file Image_10.tif]

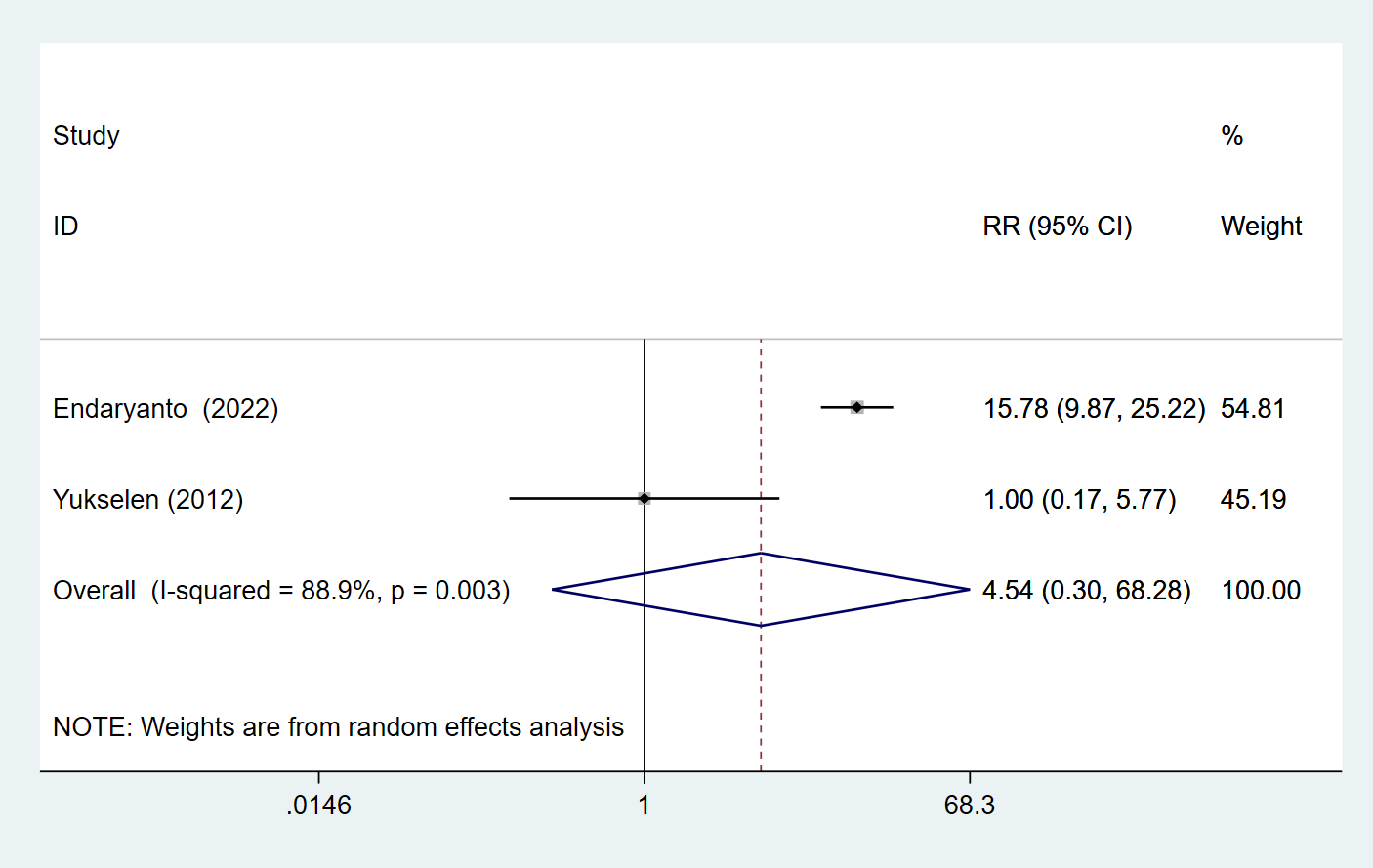

Supplement: Supplementary Figure 11 — Forest plot for TRAEs in children receiving SCIT versus non-SCIT treatment. SCIT, subcutaneous immunotherapy; RR, relative risk; CI, confidence interval; TRAEs, treatment-related adverse events. [file Image_11.tif]
