# Supplementary material for: Efficacy and safety of sublingual versus subcutaneous immunotherapy in children with allergic rhinitis: a systematic review and meta-analysis
Source: Front Immunol. 2023 Dec 15;14:1274241. doi: 10.3389/fimmu.2023.1274241 (PMC10757840; doi:10.3389/fimmu.2023.1274241)
Supplement: Supplementary file 12 [file Table_1.docx]

**Supplementary Table S1** Characteristics of the included studies.

| Author | Year | Country | Study design | AR diagnose | Group | Group division | Treatment | Sample size | Sex (M/F) | Age, years | Duration of AR, years | Allergen | Mono-/poly-sensitization status | AIT modality, | AIT protocol | Product type/ name (manufacturer) | Comorbidity | Treatment duration, months | Dropout  rate, % | QA | Outcome |
| --- | --- | --- | --- | --- | --- | --- | --- | --- | --- | --- | --- | --- | --- | --- | --- | --- | --- | --- | --- | --- | --- |
| Liu | 2023 | China | Prospective cohort | ARIA guidelines and SPT positive for Der p and specific serum IgE against Der p | SCIT | - | Subcutaneous injections of the aluminum-formulated Der p Alutard SQ vaccine; followed the recommended up-dosing schedule of 16 weeks before reaching a maintenance dose of 100,000 Alutard SQ | 44 | 34/10 | 7.11±2.04 | - | HDM | - | Injection | Continuous | Alutard SQ (ALK-Abello A/S, Horsholm, Denmark) | Asthma 28 | 24 | - | 6 | SS, MS |
|  |  |  |  |  | Non-SCIT |  | - | 11 | 6/5 | 6.83±2.23 |  |  |  | - |  | - | Asthma 7 |  |  |  |  |
| Wang | 2023 | China | RCT | Presented with a characteristic history of watery nasal discharge, nasal obstruction, sneezing, itching in the nose, positive for IgE specific to antigens such as HDM; wheal diameter ≥ 2 mm in the SPT test or the detection value of specific IgE greater than 3.5 IU/mL considered a positive result to common inhalant allergens (dust mites, pets, molds, cockroaches) | SLIT | Random | HDM allergen extract; increasing doses (No. 1, 1 mg/mL; No. 2, 10 mg/mL; No. 3, 100 mg/mL) for 3 weeks at first, receive three drops once daily (No. 4, 333 mg/mL; No. 5, 1000 mg/mL) since week 4 | 40 | 21/19 | 8.1±1.3 | ≥2 | HDM | Mono-sensitized | Drops | Continuous | Chanllergen (Wolwo Pharma Biotechnology Company, Zhejiang, China) | - | 24 | 7.5 | 4 | SS |
|  |  |  |  |  | Placebo |  | Glycerin saline solution (50 mg/mL), consisted of 50% saline buffer and 50% glycerol | 30 | 17/13 | 7.9±1.1 |  |  |  |  |  | - |  |  | 6.7 |  |  |
| Özdoğru | 2022 | Turkey | Retrospective cohort | - | SLIT | Medical records | - | 63 | - | 3-14 | - | Mixed | - | Drops | - | Stalloral 300® drops (Stallergenes®, France) | Asthma 50 | ≥36 | - | 6 | Improvement |
|  |  |  |  |  | SCIT |  |  | 45 |  |  |  |  |  | Injection |  | Novo-Helisen Depot® (Allergopharma, Germany), Phostal® (Stallergenes, France), Alutard SQ® (ALK-Abello, Denmark) | Asthma 29 |  |  |  |  |
| Endaryanto | 2022 | Indonesia | Retrospective cohort | ARIA guidelines 2016 | SCIT | - | SCIT HDM + standard treatment; 11.3–26.6 ng/mL via subcutaneous injection, the build-up phase consists of injections given weekly, in the maintenance phase, injections every three weeks; the dose of immunotherapy used every week varies from 0.1 cc (first week) to 0.15 cc (second week), 0.22 cc (third week), 0.32 cc (fourth week), 0.48 cc (fifth week), 0.72 cc (sixth week), 1 cc (seventh week), 0.1 cc (eighth week), 0.15 cc (ninth week), 0.22 cc (tenth week), 0.32 cc (eleventh week), 0.48 cc (twelfth week), 0.72 ccs (thirteenth week), and 1 cc (fourteenth week), and the following week | 1098 | 696/402 | 5.5±3.5 | - | HDM | - | Injection | Continuous | Dermatophagoides pteronyssinus extract (Teaching Industry Allergen by Airlangga University—Dr. Soetomo General Academic Hospital, Surabaya, Indonesia) | Asthma 593, bronchitis 431, atopic dermatitis 100, sinusitis 12, conjunctivitis 3, GI Problem 7, urticaria 14 | 18 | - | 7 | TRAE |
|  |  |  |  |  | Non-SCIT |  | Standard treatment | 1098 | 704/394 | 5.4±3.3 |  |  |  | - |  | - | Asthma 396, bronchitis 700, atopic dermatitis 85, sinusitis 106, GI Problem 3, urticaria 20 |  |  |  |  |
| Demoly | 2021 | Multiple countries | DBRCT | Physician-diagnosed, HDM-induced AR for at least the 12 months before inclusion in the study; SPT wheal diameter ≥ 5 mm, HDM-specific serum IgE level ≥ 3.5 kU/L | SLIT | Random | HDM AIT (a sublingual tablet formulation of standardized, purified, freeze-dried, sieved Dpte and Dfar extracts for daily administration); during the dose escalation phase consisted of a 100 IR tablet on day 1, two 100 IR tablets on day 2, and a 300 IR tablet on day 3, maintenance treatment consisted of a 300 IR tablet once daily | 155 | 91/64 | 14.1±1.75 | 5.29±3.55 | HDM | Mono-sensitized 104, poly-sensitized 51 | Tablets | Continuous | 300 IR tablet | Asthma 80 | 12 | - | 6 | TRAE |
|  |  |  |  |  | Placebo |  | Placebo | 157 | 99/58 | 14.2±1.72 | 5.27±3.39 |  | Mono-sensitized 95, poly-sensitized 62 |  |  | - | Asthma 76 |  |  |  |  |
| Endaryanto | 2021 | Indonesia | Retrospective cohort | ARIA guidelines 2016 | SCIT | - | SCIT HDM + standard treatment; 11.3–26.6 ng/mL via subcutaneous injection, 0.1 cc (first week), 0.15 cc (second week), 0.22 cc (third week), 0.32 cc (fourth week), 0.48 cc (week fifth), 0.72 cc (sixth week), 1 cc (seventh week), 0.1 cc (eighth week), 0.15 cc (ninth week), 0.22 cc (tenth week), 0.32 cc (eleventh week), 0.48 cc (twelfth week), 0.72 cc (thirteenth week), 1 cc (fourteenth week), and the following week | 1098 | 696/402 | 3-18 | - | HDM | - | Injection | Continuous | Dermatophagoides pteronyssinus extract (Teaching Industry Allergen by Dr. Soetomo Hospital-Airlangga University , Surabaya, Indonesia) | Asthma 593, bronchitis 431, atopic dermatitis 100, sinusitis 12, conjunctivitis 3, GI problem 7, urticaria 14 | 18 | - | 7 | SS, MS, SMS |
|  |  |  |  |  | Non-SCIT |  | Standard treatment, including antihistamines, intranasal steroids, and systemic steroids; bronchodilators, skin care, and physiotherapy according to the symptoms of other accompanying allergic diseases | 1098 | 704/394 |  |  |  |  | - |  | - | Asthma 396, bronchitis 700, atopic dermatitis 85, sinusitis 106, GI problem 3, urticaria 20 |  |  |  |  |
| Kim | 2021 | Korea | Retrospective cohort | SPT > 3 mm to 36 common aeroallergens | SCIT + PT | - | Subcutaneous injection of standardized extracts of up to 4 allergens + ICS + SABA; for the first 4 months, a weekly injection beginning with a 0.1 mL, doubled in strength each subsequent injection (i.e., 0.2 mL, 0.4 mL, 0.8 mL), after 4 months, monthly injections for the remainder of the treatment period (i.e., total treatment period was approximately 3 years) | 53 | 38/15 | 8 (6–15) | - | Mixed | - | Injection | Continuous | Standardized extracts of up to 4 allergens (Allerpha International, Seoul, Korea) | Asthma | 36 | - | 6 | New sensitization, improvement |
|  |  |  |  |  | PT |  | ICS + SABA | 19 | 10/9 | 9 (6–15) |  |  |  | - |  | - |  |  |  |  |  |
| Liu | 2021 | China | Prospective cohort | SPT > 3 mm and IgE>0.35 kIU/L | SLIT | Parental preference | Der f drops in increasing concentrations: No. 1, 1 μg/mL; No. 2, 10 μg/mL; No. 3, 100 μg/mL; and No.4, 333 μg/mL; during a 3-week up-dosing phase, 1, 2, 3, 4, 6, 8, 10 drops of Nos. 1-3 drops day after day in one week, after achieving the maintenance dose, three drops of No. 4 drops until the end of treatment | 125 | 52/73 | 8.5±3.1 | 2.1±1.3 | HDM | Mono-sensitized | Drops | Continuous | Der f drops (Zhejiang Wolwo Bio-Pharmaceutical Co., Ltd) | - | 24 | 36 | 5 | SS, MS, SMS, TRAE |
|  |  |  |  |  | SCIT |  | In the Alutard group, weekly injections at increasing doses of 0.2, 0.4, and 0.8 mL in the No. 1 to 3 vials and 0.1, 0.2, 0.4, 0.6, 0.8, and 1.0 mL in the No.4 vial (100 000 SQ-U/mL) during the 15-week up-dosing stage, the maintenance dose administered on a 6-week basis;  in the NHD group, injections weekly at increasing doses of 0.2, 0.4, and 0.8 mL in the No. 1-2 vials and 0.1, 0.2, 0.4, 0.6, 0.8, and 1.0 mL in the No. 3 vial (5000 TU/mL) in 18 weeks, the maintenance dose administered on a 4- to 6-week basis | 200 | 95/105 | 10.0±3.4 | 2.1±1.4 |  |  | Injection |  | Alutard SQ (ALK) or NovoHelisen Depot [NHD] (Allergopharma) |  |  | 20 |  |  |
| Wang | 2021 | China | Retrospective cohort | The symptoms of nasal congestion, sneezing, water-like snot, or rhinocnesmus, and combined with SPT or IgE positivity | SLIT + PT | Parental preference | Conventional SLIT of standardized dust mite drops + PT; in the increasing dose period (weeks 1-3), Changdi® No. 1–3 applied (protein concentrations of 1, 10, and 100 μg/mL, respectively; during a week the doses of 1, 2, 3, 4, 6, 8, and 10 drops, respectively, once per day), Changdi® No. 4 administrated starting on the 4th week (protein concentration of 333 μg/mL, once per day, 3 drops/administration, sublingual administration) | 40 | 24/16 | 8.1±2.5 | - | HDM | - | Drops | Continuous | No 1-4 Changdi® (Wolw Pharmaceuticals, Zhejiang, China) | - | 12 | - | 6 | SS, MS |
|  |  |  |  |  | PT |  | Conventional PT, including the second-generation antihistamines (oral, once per day) and nasal corticosteroids (nasal spray, 1–2 times per day) | 40 | 27/13 | 7.8±2.5 |  |  |  | - |  | - |  |  |  |  |  |
| Chen | 2020 | China | RCT | SPT for D. farinae and/or D. pteronyssinus showing a positive reaction (at least ++ or above) | SLIT + PT | Random | Standardized D. farinae drops + PT | 55 | 35/20 | 6.8 ± 0.34 | ≥1 | HDM | Mono-sensitized 27, poly-sensitized 28 | Drops | Continuous | Chanllergen™ (Zhejiang Wolwo Biopharmaceutical Co., Ltd, Zhejiang, China) | - | 36 | 26.67 | 2 | SS, MS, SMS |
|  |  |  |  |  | PT |  | PT only | 47 | 30/17 | 6.1 ± 0.35 |  |  | - | - |  | - |  |  | 37.33 |  |  |
| Nolte | 2020 | Multiple countries | DBRCT | Physician diagnosis of AR/C (with or without asthma), treatment for ragweed polleneinduced AR/C during the previous ragweed pollen season, a positive SPT result to A artemisiifolia (average wheal diameter ≥5 mm than with saline control) and specific IgE against A artemisiifolia ≥ 0.7 kUA/L (ImmunoCAP singleplex, ThermoFisher Scientific) at screening | SLIT | Random | Once-daily treatment with the 12 Amb a 1-Unit dose of ragweed SLIT-tablet | 512 | 324/188 | 12.1 ± 3.2 | - | Ragweed pollen | Mono-sensitized 107, poly-sensitized 405 | Tablets | Pre-and coseasonal | Ragwitek/Ragwizax (ALK-Abelló, Hørsholm, Denmark) | Asthma 219 | 5.7 | 10.00 | 6 | SS, MS, SMS, TRAE |
|  |  |  |  |  | Placebo |  | Placebo | 510 | 319/191 | 12.2 ± 3.1 |  |  | Mono-sensitized 121, poly-sensitized 389 |  |  | - | Asthma 217 | 5.8 | 3.70 |  |  |
| Proctor | 2020 | UK | Retrospective cohort | SPT and/or specific IgE levels | Pollen SCIT | Parental preference | Pollinex quattro SCIT; pre-seasonally as four injections at weekly intervals for 3 years in the day-case unit | 113 | 77/36 | 15 (12-15)* | - | HDM, pollen | Mono-sensitized 56, poly-sensitized 57 | Injection | Preseasonal | Pollinex quattro SCIT (Allergy Therapeutics, London, UK) | Asthma 51, eczema 10, asthma+eczema 31 | 36 | 13 | 5 | SS |
|  |  |  |  |  | Pollen SLIT |  | Grass pollen or HDM SLIT; daily sublingual tablets or drops | 42 | 30/12 | 11 (9-14)* |  |  | Mono-sensitized 19, poly-sensitized 23 | Tablets or drops | Continuous | Grazax® (ALK-Abello, Reading, UK) | Asthma 13, eczema 2, asthma+eczema 6 |  | 19 |  |  |
|  |  |  |  |  | HDM SLIT |  |  | 94 | 56/38 | 12 (10-15)* |  |  | Mono-sensitized 23, poly-sensitized 71 |  |  | Oralvac® (Allergy therapeutics, Worthing, UK) | Asthma 37, eczema 9, asthma+eczema 36 |  | 28 |  |  |
| Biedermann | 2019 | Multiple countries | DBRCT | A positive SPT response (wheal diameter≥3 mm) to birch, a positive Bet v 1–specific IgE level (IgE class 2 or greater≥0.7 kU/L) | SLIT | Random | 1 daily tablet of SQ tree SLIT-tablet | 28 | - | 12-17 | - | Birch pollen | - | Tablets | Pre-and coseasonal | SLIT–tablet (ALK-Abello, Hørsholm, Denmark) | - | 6.5-9.5 | - | 6 | TRAE |
|  |  |  |  |  | Placebo |  | Placebo | 32 |  |  |  |  |  |  |  | - |  |  |  |  |  |
| Okamoto | 2019 | Japan | DBRCT | AR symptoms for ≥2 years, IgE antibody specific to D. pteron‐ yssinus and/or D. farinae antigens ≥ 3 | SLIT | Random | HDM tablets (active) once daily; dose increased from 100IR (Day 1) to 200IR (Day 2) to the maintenance dose of 300IR (Day 3 to Week 52) | 205 | 123/82 | 10.3 ± 2.7 | <5 69, 5-10 116, ≥10 20 | HDM | Mono-sensitized 39, poly-sensitized 166 | Tablets | Continuous | Actair® (Stallergenes Greer, Antony, France)) | Asthma 26 | 12 | 11.87 | 5 | SS, MS, SMS, improvement, TRAE |
|  |  |  |  |  | Placebo |  | Placebo | 217 | 136/81 | 10.4 ± 2.7 | <5 62, 5-10 137, ≥10 18 |  | Mono-sensitized 44, poly-sensitized 173 |  |  | - | Asthma 33 |  | 4.11 |  |  |
| Yu | 2019 | China | Retrospective cohort | Diagnosis and Treatment Principles and Recommendations for Allergic Rhinitis; SPT only dust mite sensitivity (positive + + or above + +) | SLIT | - | D. farinae drops by sublingual; 1 drop of D. farinae drop 1 dripped under the tongue and swallow after 3 min; D. farinae drops 2 and 3 given 2 and 3 weeks respectively, after treatment for 7 consecutive days once daily, the doses during the 7 days were 1 drop on the first day, 2 on the second day, 3 on the third day, 4 on the fourth day, 6 on the fifth day, 8 on the sixth day, and 10 on the seventh day; four weeks after treatment, D. farinae drop 4 once daily for consecutive treatment | 52 | 33/19 | 6.15±0.83 | 2.41±1.34 | HDM | Mono-sensitized | Drops | Continuous | D. farinae drops (Zhejiang Wolwo Pharma Technologies Inc., batch number: S20060012) | - | 15 | - | 6 | SS, MS, improvement |
|  |  |  |  |  | PT |  | Conventional anti-allergic drugs, including 10 mg/d ebastine orally and levocabastine by nasal spray | 50 | 29/21 | 6.34±0.91 | 2.32±1.19 |  |  |  |  | Ebastine (Jiangsu Lianhuan Pharmaceutical Group CO., LTD, batch number: H20040119) and levocabastine (Shanghai Johnson Pharmaceutical Co., Ltd., batch number: 20160072) |  |  |  |  |  |
| Masuyama | 2018 | Japan | DBRCT | A medication history for HDM AR of at least 1 year; a positive nasal provocation test to house dust; a positive specific serum IgE level (≥3.5 kU/L) against D. pteronyssinus, D. farinae, or both | SLIT | Random | Initiated daily SQ HDM SLIT‐tablet treatment at a dose of 3300 JAU for a week, from week 2 and onward, 10 000 JAU throughout the remainder of the trial | 227 | 150/77 | 10.8 ± 2.9 | 5.2±3.0 | HDM | Mono-sensitized 51, poly-sensitized 176 | Tablets | Continuous | SLIT‐tablet (TO‐203, Torii, Japan; manufactured by ALK, Denmark) | Asthma 10 | 12 | 8 | 7 | TRAE |
|  |  |  |  |  | Placebo |  | Placebo | 231 | 154/77 | 10.7 ± 3.1 | 5.2±3.5 |  | Mono-sensitized 74, poly-sensitized 157 |  |  | - | Asthma 6 |  | 6 |  |  |
| Song | 2018 | China | Cohort | ARIA guidelines and SPT positive to HDM allergens (Dermatophagoides pteronyssinus and/or Dermatophagoides farinae) | SCIT | Parental preference | Alutard SQ SCIT, weekly injections with volumes of 0.2, 0.4 and 0.8 ml from vial numbers 1-3, and 0.1, 0.2, 0.4, 0.8 and 1.0 ml from vial number 4, to achieve a maintenance dose of 100 000 SQ-U, the maintenance dose administered every 6 weeks for 36 months | 106 | 61/45 | 9.1±4.3 | 3.8±1.8 | HDM | Mono-sensitized 89, poly-sensitized 17 | Injection | Continuous | Alutard SQ (Dermatophagoides pteronyssinus; ALK-Abelló, Hørsholm, Denmark) | Asthma 13, conjunctivitis 11, urticaria 4 | 36 | - | 5 | SS |
|  |  |  |  |  | PT |  | Intranasal corticosteroids (budesonide) and oral antihistamines (loratadine) for 1 month, budesonide and loratadine treatment again, depending on the persistence and severity of the AR symptoms | 87 | 54/33 | 9.5±3.7 | 3.4±1.9 |  | Mono-sensitized 68, poly-sensitized 19 | - |  | Corticosteroids (budesonide) and antihistamines (loratadine) | Asthma 10, conjunctivitis 9, urticaria 1 |  |  |  |  |
| Valovirta | 2018 | 11 European countries | DBRCT | SPT response (wheal diameter≥3 mm) and specific IgE to Phleum pratense (≥IgE class 2, ≥0.70 kU/L); grass ARC requiring allergy PT during 2 grass pollen seasons prior to randomization | SLIT | Random | Grass SLIT tablet, 1 tablet daily | 398 | 255/143 | 8.5±2.1 | 3.5 (0.2-10) | Grass pollen | Mono-sensitized 132, poly-sensitized 266 | Tablets | After GPS | Grass SLIT tablet | Eczema 60, food allergy 55 | 36 | 25 | 5 | SS, MS, development of asthma, TRAE |
|  |  |  |  |  | Placebo |  | Placebo tablets identical to the grass SLIT tablets but without pollen extract | 414 | 256/158 | 8.7±2.1 | 3.4 (0.3-10) |  | Mono-sensitized 149, poly-sensitized 265 |  |  | - | Eczema 48, food allergy 51 |  |  |  |  |
| Chen | 2017 | China | RCT | A clinical history of HDM induced AR for at least one year and SPT confirmed | SLIT | Random | The HDM allergen extract for SLIT used in the form of drops (n°1, 1 mg/mL; n° 2, 10 mg/mL; n°3, 100 mg/mL and n° 4, 333 mg/mL), take increasing doses (from n°1 to n°3) during the first three weeks’ up-dosing phase, and then take 3 drops of n° 4 solution once daily during the maintenance phase | 21 | 10/11 | 10.1±3.7 | 5.2±2.4 | HDM | - | Drops | Continuous | HDM allergen extract (Wolwopharma Biotechnology Company, Zhejiang, China) | - | 12 | 0 | 6 | SS |
|  |  |  |  |  | Placebo |  | Diluents containing 50% glycerol and 50% saline buffer | 21 | 11/10 | 10.3±3.5 | 6.1±3.7 |  |  |  |  | - |  |  |  |  |  |
| Lim | 2017 | Korea | Retrospective cohort | Rhinitis symptoms (at least one of the nasal obstructions, rhinorrhea, sneezing, and itching sense of nose) and using SPT with inhalant allergens for allergic sensitivity | SLIT+PT | - | Standardized HDM extracts + PT; for 30 days of escalation period, increased the dose of administration as follows: 1 to 5 drops of 1.6 STU (specific treatment unit) /mL solution from days 1 to 10, 1 to 5 drops of 8 STU/mL solution from days 11 to 15, 1 to 5 drops of 40 STU/mL solution from days 16 to 20, 1 to 5 drops of 200 STU/mL solution from days 21 to 25, and 1 to 5 drops of 1000 STU/mL solution from days 26 to 30, then maintained 5 drops of 1,000 STU/mL solution, 3 times per week | 37 | 154/67 | 8.4 (5-13) | - | HDM | Mono-sensitized 22, poly-sensitized 15 | Drops | Continuous | Pangramin SLIT® (ALK-Abello, Madrid, Spain) | - | 36 | - | 6 | SS, new sensitization |
|  |  |  |  |  | PT |  | PT (oral medication and/or topical steroids) on demand alone | 184 |  | 7.6 (4-13) |  |  | Mono-sensitized 97, poly-sensitized 87 | - |  | - |  |  |  |  |  |
| Matsuoka | 2017 | North America, Japan | DBRCT | Positive SPT≥5 mm compared with saline control and serum specific IgE of ≥0.7 kU/L to either Dermatophagoides (D.) farinae or D. pteronyssinus; or a positive nasal provocation test and a serum specific IgE of ≥3.5 kU/L to either D. farinae or D. pteronyssinus. | SLIT | Random | Trial A: 12 SQ HDM once daily for 12 months trial B: 6 SQ HDM or 12 SQ HDM once daily for 12 months; 2 SQ HDM for the first week, 6 SQ HDM for the second week (and for the 6 SQ HDM group throughout the trial) and 12 SQ HDM for the third week and throughout the trial (12 SQ HDM group) | 201 | 120/81 | 14.3±1.6 | 6.6±3.6 | HDM | Mono-sensitized 45, poly-sensitized 156 | Tablets | Continuous | SQ HDM SLIT-tablet (ALK, Denmark) | Asthma 39 | 9-12 | 13 | 6 | TRAE |
|  |  |  |  |  | Placebo |  | Placebo | 194 | 106/88 | 14.5±1.7 | 7.1±3.7 |  | Mono-sensitized 44, poly-sensitized 149 |  |  | - | Asthma 40 |  | 10 |  |  |
| Wang | 2017 | China | RCT | At least two or more symptoms of sneezing, runny nose, nasal congestion and nasal itching; SPT for HDM showed strong positive reaction (at least ++ or above), as confirmed by specific IgE test (>Ⅱ level) | SLIT | Random | Single HDM extract, biologically standardized extracts labeled with five different concentrations: Der . f drops No. 1 (1 μg/mL), No. 2 (10 μg/mL), No. 3 (100 μg/mL), No. 4 (333 μg/mL) and No.5 (1000 μg/mL); increasing doses for 3 weeks at first, maintained with the Der . f drops No. 4 | 34 | 16/18 | 9.6±2.5 | - | HDM | Poly-sensitized | Drops | Continuous | Chanllergen (Wolwopharma Biotechnology Company, China) | - | 12 | 0 | 3 | SS, MS |
|  |  |  |  |  | SCIT |  | Concentrations of subcutaneous injection in the deltoid muscle of the upper arm started from 1:10000; during the conventional treatment of twice a week, the injection dosage started from 0.1 mL, increased by 0.1 mL every time until 1 mL; the next concentration level then used and repeated until three months later, the highest concentration (1:100) achieved and the maintenance therapy started; during the maintenance therapy, the allergen concentration 1:100 and each dose 0.5 mL; at first injections twice a week (total 10 times), and gradually injection intervals extended according to the efficacy, such as once a week, once two weeks, once four weeks, each time the injection interval changed, the allergen injected ten times, and injected every four weeks in the long-term maintenance therapy | 34 | 17/17 | 9.4±2.4 |  |  |  | Injection |  | Subcutaneous desensitization agents (New-Hualian Union Pharmaceutical Company, China) |  |  |  |  |  |
| Luo | 2016 | China | Cohort | Clinical criteria of moderate to severe persistent AR, D. pteronyssinus and/or D. farinae with a positive SPT (wheal diameter >6 mm) and/or a CAP-Pharmacia score >class 2 | SLIT | - | HDM allergen extract; labeled with the concentration of total protein and in the form of drops (No. 1, 1 μg/mL; No. 2, 10 μg/mL; No. 3, 100 μg/ mL; and No. 4, 333 μg/mL) | 11 | 14/10 | 6.5±2.7 | 2.9±1.4 | HDM | Mono-sensitized 22, poly-sensitized 2 | Drops | - | Chanllergen (Wolwopharma Biotechnology Company, Zhejiang, China) | Asthma 2 | 3 | 0 | 5 | SS |
|  |  |  |  |  | SCIT |  | A standardized mite depot-allergen extract (50% D. pteronyssinus and 50% D. farinae) | 13 |  |  |  |  |  | Injection |  | mite depot-allergen extract (Allergopharma Joachim Ganzer KG, Reinbek, Germany) |  |  |  |  |  |
| Maloney | 2016 | USA | DBRCT | A clinical history of HDMinduced AR/C (diagnosed by a physician) of at least 6 months’ duration, a positive SPT reaction (wheal diameter ≥5 mm larger than saline control) against D pteronyssinus or D farinae, serum specific IgE against D pteronyssinus or D farinae of at least 0.7 kU/L or at least class 2, have a forced expiratory volume in 1 second of at least 70% of predicted | SLIT | Random | HDM SLIT tablet 6 SQ-HDM or 12 SQ-HDM once daily for 28 days | 130 | 78/52 | 14.5±1.6 | 8.7±3.8 | HDM | Mono-sensitized 27, poly-sensitized 103 | Tablets | - | MK-8237 (Merck, ALK Abellò, and Torii) | Asthma 46 | 1 | 6.92 | 6 | TRAE |
|  |  |  |  |  | Placebo |  | Placebo | 65 | 44/21 | 14.3±1.8 | 8.2±4.5 |  | Mono-sensitized 12, poly-sensitized 53 |  |  | - | Asthma 22 |  | 0 |  |  |
| Wang | 2016 | China | DBRCT | SPT ≥ 2 mm or specific IgE≥ 0.35 IU/mL | SLIT | Random | HDM allergen extract in the form of drops (no. 1, 1 mg/mL; no. 2, 10 mg/mL; no. 3, 100 mg/mL and no. 4, 333 mg/mL), take increasing doses (from no. 1 to 3) during the first three weeks up-dosing phase, then have 3 drops of no. 4 solution once daily during the maintenance phase | 25 | 11/14 | 6-14 | 4.3±2.1 | HDM | Mono-sensitized | Drops | Continuous | Chanllergen (Wolwopharma Biotechnology Company, Zhejiang, China) | - | 12 | 0 | 6 | SS, MS |
|  |  |  |  |  | Placebo |  | A diluent containing 50% glycerol and 50% saline buffer | 25 | 12/13 | 6-16 | 3.6±1.8 |  |  |  |  | - |  |  |  |  |  |
| Yin | 2016 | China | RCT | SPT positive | SLIT + PT | Random | Sublingual administration of dust mite drops + standard GINA schemes; solutions 1 to 3 (1, 10, 100mg/l) used at the initial increasing phrase, and 4 to 5 ( 333 and 100 mg/l)at a later maintenance phase; treatment began with the solution 1, administering one drop under the tongue and then swallowing and then repeating as appropriate, using the medicine once per day at the same time point in the morning on an empty stomach or before going to bed; the doses gradually increased daily (doses of 1st to 7th day were 1, 2, 3, 4, 6, 8 and 10 drops respectively); solution 2 used in the second week, solution 3 the third week, finally the maintenance phase began from the forth week on, at this point patients took three drops of solution 4 once before sleep, patients over 12 years of age took solution 5 to maintain treatments | 78 | 42/36 | 11.2±4.8 | 0.30+0.1 | HDM | - | Drops | Continuous | Chang Di (Zhejiang WOWU Biology Co., Ltd., Shanghai, China) | Asthma | 24 | - | 3 | SS |
|  |  |  |  |  | PT |  | Standard GINA asthma control schemes, using intravenous drip, inhaled or oral hormones, leukotriene receptor antagonists, antihistamines, bronchial beta agonists, theophylline and so on the basis of disease severity | 78 | 40/38 | 10.3±4.5 | 0.28+0.1 |  |  | - |  | - |  |  |  |  |  |
| Shao | 2014 | China | RCT | Have a clinical history of mite allergy and sensitization to Dermatophagoides farinae as confirmed by a SPT and serum-specific IgE of≥0.7 kU/L, and/or a forced expiratory volume in 1 second of≥70% of predicted volume | SLIT + PT | Random | Standardized D. farinae drops; during first 3 weeks, started on day 1 with 0.05 ml, the dose increased by 0.05 ml per day until day 4, the rest 3 days increased by 0.10 ml per day, then the maintenance dose 0.15 ml | 168 | 104/64 | 6.37±2.59 | - | HDM | Mono-sensitized 7 poly-sensitized 161 | Drops | Continuous | Standardized D. farinae drops (Zhejiang Wolwo Bio-Pharmaceutical Co., Ltd.) | Asthma 139, conjunctivitis 4, atopic dermatitis 3, food allergy 17 | 12 | 16.07 | 2 | New sensitization, TRAE |
|  |  |  |  |  | PT |  | Standardized PT | 96 | 59/37 | 5.92±3.04 |  |  | Mono-sensitized 1 poly-sensitized 95 | - |  | - | Asthma 79, conjunctivitis 1, food allergy 1 |  | 19.79 |  |  |
| Aydogan | 2013 | Turkey | DBRCT | SPT positivity (≥ 3 mm) to HDM | SLIT | Random | Standardized extract; daily increasing doses in the form of 1e10 drops of the 10 IR/mL extract during the first 6 days; for reaching maintenance dose, 300 IR/mL concentration vials initiated on the 7th day starting from 1 and reaching to 8 drops on the 11th day; then took 8 drops sublingually 3 times a week for 12 months | 7 | 6/1 | 8.1±2.2 | 1.9±1.5 | HDM | Mono-sensitized | Drops | Continuous | STALORAL®(Stallergenes SA, Antony, France) | - | 12 | 12.5 | 4 | SS, MS, TRAE |
|  |  |  |  |  | Placebo |  | Placebo | 9 | 6/3 | 7.3±2.3 | 1.7±1.4 |  |  |  |  | - |  |  | 10 |  |  |
| de Bot | 2012 | The Netherland | DBRCT | IgE antibodies ≥0.7 kU/l to HDM, had a retrospective nose symptom score of at least 4 of 12 points during the last 3 months | SLIT | Random | Received an aqueous extract of HDM (D. pter, Dermatophagoides pteronyssinus) in a glycerinated isotonic phosphate-buffered solution; started on day 1 with 0.05 ml (1 drop) corresponding with 35 BU, the dose increased by 1 drop per day until day 20 (20 drops = 1 ml = 700 BU), the maintenance dose 20 drops twice weekly after dose escalation phase | 110 | 67/43 | 11.8 ± 3.1 | ≥1 | HDM | Mono-sensitized 16, poly-sensitized 94 | Drops | Continuous | Oralgen Mijten® (Artu Biologicals, Lelystad, The Netherlands) | Wheeze or dyspnea 60 | 24 | 13.6 | 4 | SS, improvement |
|  |  |  |  |  | Placebo |  | Placebo consisting of the glycerol-containing solvent | 116 | 68/48 | 11.7 ± 2.9 |  |  | Mono-sensitized 28, poly-sensitized 88 |  |  | - | Wheeze or dyspnea 63 |  | 11.9 |  |  |
| Stelmach | 2012 | Poland | DBRCT | Positive SPT and specific IgE | Continuous SLIT | Random | Standardized extract of five grass pollen; on the average of 8 weeks before the pollen season, 1 drop on first day followed by 3, 5, 7, and 12 drops (four puffs) on each consecutive day, in the maintenance phase every morning before breakfast, four puffs from a dispenser, active drug (10 μg of major allergens) for 12 months | 19 | 14/5 | 10.1 (3–16) | - | Grass pollen | Mono-sensitized | Drops | Continuous | Staloral 300 IR (Stallergenes SA, Antony, France) | Asthma 5 | 24 | 5 | 6 | SS, MS, SMS, TRAE |
|  |  |  |  |  | Pre-coseasonal SLIT |  | Standardized extract of five grass pollen; on the average of 8 weeks before the pollen season, 1 drop on first day followed by 3, 5, 7, and 12 drops (four puffs) on each consecutive day, in the maintenance phase every morning before breakfast, four puffs from a dispenser, active drug (10 μg of major allergens) for 6 months followed by 6 months of placebo | 17 | 11/6 | 8.3 (5–17) |  |  |  |  | Pre-and coseasonal |  | Asthma 6 |  | 15 |  |  |
|  |  |  |  |  | Placebo |  | Identically looking verum dispensed in the same glycerosaline diluents | 18 | 11/7 | 8.1 (4–15) |  |  |  |  | - | - | Asthma 5 |  | 10 |  |  |
| Wahn | 2012 | Germany, Poland | DBRCT | IgE-mediated allergic rhinitis/rhinoconjunctivitis; grass pollen sensitivity, documented by a SPT at least as large as the histamine-di-hydrochloride (0.1 %) reaction or >5 mm in diameter; positive enzyme allergosorbent test (EAST) > class 2 to grass pollens; proven clinical relevance by positive conjunctival provocation testing with grass allergens | SLIT | Random | An aqueous grass pollen preparation containing 6 species in equal amounts in a water/glycerol solution with phosphate-buffered saline; 3600 to 4800 mg of grass group 5 until the first grass pollen season and 3600 mg grass group 5 during the pollen season (May to July) | 158 | 111/47 | 8.74±2.27 | - | Grass pollen | - | Drops | Pre-and coseasonal | Aqueous grass pollen preparation(Allergopharma Joachim Ganzer KG, Reinbek, Germany ) | Asthma 49 | 8 | 16.46 | 5 | SS, MS, SMS, TRAE |
|  |  |  |  |  | Placebo |  | A water/glycerol solution with phosphate-buffered saline | 49 | 35/14 | 8.67±2.32 |  |  |  |  |  | - | Asthma 15 |  | 4.08 |  |  |
| Yukselen | 2012 | Turkey | DBRCT | Criteria in the WHO consensus statement on ARIA, verified by a positive SPT (a wheal> 3 mm) and the presence of specific IgE (sIgE; radioallergosorbent test, RAST, class II or > 0.70 kU/l) | SCIT | Random | Active subcutaneous immunotherapy (injections) and placebo sublingual drops; a 12-week induction phase (weekly injections) starting with a dose of 0.2-0.8 ml of 50 TU/ml (weeks 1-3), 0.2-0.8 ml of 500 TU/ml (weeks 4–8) and 0.2-0.8 ml of 5,000 TU/ml (weeks 9–12); the maximum tolerated dose achieved during the induction phase was the maintenance dose, and repeated every fourth week, the cumulative 1-year dose approximately 43,770 TU (21,885 of TU D.pt. and 21885 TU of D.f.) | 10 | 6/4 | 10.9±3.2 | 6 (3-12) | HDM | Mono-sensitized | Injection | Continuous | NovoHelisen Oral (Allergopharma) | Asthma | 12 | 0.00 | 6 | SS, MS, TRAE |
|  |  |  |  |  | SLIT |  | Active sublingual immunotherapy (drops) and placebo subcutaneous injections; taken drops before meals daily, increased for 12 weeks until the maintenance dose reached, initial dose 1 drop of 10 TU/ml increasing to 28 drops on day 28, 1–28 drops of 100 TU/ml on days 29–56 and 1–28 drops of 1,000 TU/ ml on days 57–84; after reached this dose or the maximum tolerated dose, the maintenance dose administered 3 times per week as 28 drops of 1,000 TU/ml, the cumulative 1-year dose approximately 173,733 TU (86,866.5 TU of D.pt. and 86,866.5 TU of D.f.) | 10 | 5/5 | 9.2±3.4 | 4.5 (1-12) |  |  | Drops |  | NovoHelisen Depot (Allergopharma) |  |  | 9.09 |  |  |
|  |  |  |  |  | Placebo |  | Placebo sublingual drops and placebo subcutaneous injections | 10 | 6/4 | 10.1±2.7 | 5.5 (3-8) |  |  | Mixed |  | - |  |  | 0.00 |  |  |
| Blaiss | 2011 | USA, Canada | DBRCT | Treatment for ARC during the previous grass pollen season; a positive SPT response to P pratense with the average of the horizontal and vertical wheal diameters 5 mm or larger than that elicited by the saline control, a positive specific IgE level against P pratense of 0.7 kU/L or greater | SLIT | Random | Grass AIT; once daily sublingual dose of 2800 bioequivalent allergen units | 175 | 118/57 | 12.1 (6-17) | - | Grass pollen | Mono-sensitized 23, poly-sensitized 152 | Tablets | Pre-and coseasonal | Oral lyophilisate (Phleum pratense, Schering-Plough Corp, a division of Merck & Co, Kenilworth, NJ) | Asthma 46 | 5.8 | 18.86 | 5 | SS, MS, SMS, TRAE |
|  |  |  |  |  | Placebo |  | Identical in composition and physical properties to active treatment but with no grass pollen extract included | 169 | 105/64 | 12.6 (5-18) |  |  | Mono-sensitized 15, poly-sensitized 154 |  |  | - | Asthma 44 |  | 17.16 |  |  |
| Yonekura | 2010 | Japan | DBRCT | clinical history, positive allergen-specific SPT (wheal diameter ≥10 mm) to house dust extract, and a serum HDM-specific IgE score ≥2 on the CAP-radioallergosorbent test | SLIT | Random | House dust extract; following a week for washout before treatment (Week 0), the dose escalated over a period of 3 weeks by administration of an increasing number of extract drops at three concentrations, increasing doses from each vial, beginning with 0.2 ml from a 1000-fold diluted vial, and increasing by 0.2 ml per day over 5 days, then repeated with each vial until the maximum dose (1.0 ml of a 10-fold diluted vial) reached | 19 | 13/6 | 9.4±2.2 | 5.9±2.9 | HDM | - | Drops | - | Extracts of house dust (Torii Pharmaceutical: lot number; ASCY) | Bronchial asthma 8, atopic dermatitis 5, Japanese cedar pollinosis 1 | 10 | 5.00 | 6 | TRAE, improvement |
|  |  |  |  |  | Placebo |  | Placebo | 9 | 7/2 | 9.6±2.5 | 5.3±2.1 |  |  |  |  | - | Bronchial asthma 7, atopic dermatitis 2, Japanese cedar pollinosis 1 |  | 18.18 |  |  |
| Acquistapace | 2009 | Italy | Retrospective cohort | ARIA guidelines | SLIT | - | Specific SLIT with the related allergen extracts once daily | 90 | 65/25 | 11±3 | ≥2 | Mixed | Mono-sensitized 36, poly-sensitized 54 | Drops | Continuous | SLITone® (ALK-Abello` , Lainate, Italy) | - | 24 | - | 6 | SS, MS, new sensitization, development of asthma |
|  |  |  |  |  | Non-SLIT |  | Never been treated with specific immunotherapy | 81 | 59/22 | 12±3 |  |  | Mono-sensitized 34, poly-sensitized 47 | - |  | - |  |  |  |  |  |
| Bufe | 2009 | Germany | DBRCT | Positive SPT against P pratense (wheal diameter >3 mm), specific IgE against P pratense (IgE class ≥2) | SLIT | Random | An orodispersible, fast-dissolving, SQ-standardized grass allergen tablet once daily | 126 | 83/43 | 10.1±2.9 | 3.5±2.6 | Grass pollen | Mono-sensitized 24, poly-sensitized 102 | Tablets | Pre-and coseasonal | Grazax (ALK, Hørsholm, Denmark) | Asthma 55 | 3.5-10.4 | 9.52 | 5 | SS, MS, TRAE |
|  |  |  |  |  | Placebo |  | Placebo tablet similar in taste, smell, and appearance, but contained no histamine or any other active ingredients | 127 | 83/44 | 10.1±3.1 | 3.4±2.4 |  | Mono-sensitized 21, poly-sensitized 106 |  |  | - | Asthma 50 |  | 5.51 |  |  |
| Wahn | 2009 | Germany, Denmark, Poland, France, Spain | DBRCT | A positive SPT response (wheal diameter >3 mm) and a timothy grass pollen–specific IgE level of at least class 2 (≥0.7 kU/L) | SLIT | Random | Once-daily SLIT with 300 IR of allergen extract in a tablet formulation, increased by 100 IR per day over 3 days | 131 | 86/45 | 10.5±3.34 | ≥2 | Grass pollen | Mono-sensitized 54, poly-sensitized 77 | Tablets | Pre-and coseasonal | Freeze-dried allergen extract of 5 grass pollens (Stallergenes, Antony, France) | Asthma 28 | 5.0±0.6 | 2.88 | 6 | SS, MS, TRAE |
|  |  |  |  |  | Placebo |  | Placebo | 135 | 85/50 | 11.2±3.07 |  |  | Mono-sensitized 55, poly-sensitized 80 |  |  | - | Asthma 29 |  | 5.76 |  |  |
| Marogna | 2008 | Italy | RCT | SPT>5 mm | SLIT + PT | Random | SLIT for the responsible allergen + drug therapy; prescribed SLIT prepared as a glycerinated solution administered as sublingual drops in the morning, the build-up phase (approximately 50 days) involved the administration of increasing concentrations (100-3000 RU/mL), in the maintenance phase, 5 drops from the 10,000 RU/mL vial given 3 times a week, a dose reduction by one-third in the pollen season | 144 | 104/40 | 10.7±0.43 | ≥2 | HDM 98, pollen 46 | Mono-sensitized 81, poly-sensitized 63 | Drops | Continuous | Prescribed SLIT (Anallergo, Florence, Italy) | Asthma 86 | 36 | 9.72 | 4 | New sensitization, development of asthma |
|  |  |  |  |  | PT |  | Standard drug therapy | 72 | 43/29 | 10.0±0.3 |  | HDM 48, pollen 24 | Mono-sensitized 38, poly-sensitized 34 | - |  | - | Asthma 45 |  | 8.33 |  |  |
| Tseng | 2008 | China | DBRCT | Based on history, a positive SPT for HDM, and the presence of serum specific IgE (≥ 3 on the MAST CLA allergen test) | SLIT | Random | A standardized extract of equal amounts of allergens from the HDM; during the 3-week induction period, used an accelerated dosing schedule, for the first week, the SLIT vial contained 10 IR/ml and start with one drop and gradually increase to 10 drops by day 7, switched to the second-week vial containing 100 IR/ml, start with 1 drop per day and gradually increase the dose to 20 drops on day 14, the third-week and subsequent maintenance vials all contained Staloral 300 IR/ml, on day 15, take 7 drops and increase the dose to 20 drops on day 19, then used for another 21 weeks, If taken as directed, the cumulative dose would be 37,312 IR, equivalent to 1.56 mg of D.p. and 2.71 mg of D.f | 30 | 22/8 | 9.7±3.3 | 2-5 19, 6-10 10, >10 1 | HDM | Mono-sensitized | Drops | - | Staloral (Stallergenes, France) | - | 6 | 6.67 | 5 | SS, MS, TRAE |
|  |  |  |  |  | Placebo |  | Placebo glycerol saline diluent | 33 | 23/10 | 9.7±3.0 | 2-5 17, 6-10 16 |  |  |  |  | - |  |  | 6.06 |  |  |
| Ibanez | 2007 | Germany, Spain | DBRCT | Positive specific IgE against P. pratense (≥0.7 kU/ml); positive SPT against P. pratense wheal diameter ≥3 mm | SLIT | Random | Grazax tablet once daily | 45 | 28/17 | 8.4±2.2 | 2.8±1.3 | Grass pollen | - | Tablets | Outside the GPS | Grazax® (ALK, Hørsholm, Denmark) | Asthma 18 | 1 | 6.67 | 5 | TRAE |
|  |  |  |  |  | Placebo |  | Placebo | 15 | 12/3 | 9.2±2.6 | 3.3±1.7 |  |  |  |  | - | Asthma 6 |  | 0.00 |  |  |
| Jacobsen | 2007 | Multiple countries | RCT | Had a clinical history of birch and/or grass pollen induced seasonal allergic rhinoconjunctivitis, positive SPT and CPT results | SCIT | Random | Standardized allergen extracts of grass pollen (Phleum pratense) and/or birch pollen (Betula verrucosa); weekly injections over 15–20 weeks,maintenance injections with the depot preparation given every 6 weeks (±2 weeks) for a total period of 3 years,the contents of major allergen per maintenance injection (Alutard SQ 100 000 SQ units/ ml) corresponded to 20 lg Phl p 5 (grass) and 12 lg Bet v 1 (birch) | 103 | 137/68 | 6-14 | - | Birch and/or grass pollen | - | Injection | Continuous | Alutard SQ (ALK-Abello, Horsholm, Denmark) or Aquagen SQ (ALK-Abello) | - | 36 | 23.30 | 2 | Development of asthma |
|  |  |  |  |  | Non-SCIT |  | Standard drug therapy | 102 |  |  |  |  |  | - |  | - |  |  | 33.33 |  |  |
| Röder | 2007 | The Netherlands | DBRCT | IgE antibodies to grass pollen ≥0.7 kU/L and a history of rhinoconjunctivitis | SLIT | Random | Verum treatment with a mixture of aqueous extracts of 5 grass pollen species in a glycerinated isotonic phosphate-buffered solution; starting with a single drop containing 475 biological units(BU) of allergen increased with 1 drop daily until day 20, the maintenance dose 20 drops (9,500 BU; 21 mg equivalent Lol p 5) twice weekly for 2 years, resulting in a mean cumulative dose of 1,976,000 BU (4.5 mg equivalent Lol p 5) | 91 | 61/30 | 12.9±2.6 | - | Grass pollen | Mono-sensitized 22, poly-sensitized 69 | Drops | After GPS | Oralgen Grass Pollen (Artu Biologicals, Lelystad, The Netherlands) | Lower airway symptoms 52 | 24 | 24.07 | 6 | SS |
|  |  |  |  |  | Placebo |  | Underwent placebo consisted of the solvent | 77 | 34/43 | 12.5±2.9 |  |  | Mono-sensitized 20, poly-sensitized 57 |  |  | - | Lower airway symptoms 46 |  | 25.00 |  |  |
| Eng | 2006 | Switzerland | Prospective cohort | With a history of severe grass pollen allergic rhinoconjunctivitis for at least 2 years but with IgE-mediated sensitivity to seasonal allergens | SCIT + PT | - | Grass pollen depotallergoids in a preseasonal immunotherapy protocol | 14 | 10/4 | 9.5 (5–16) | ≥2 | Grass pollen | - | Injection | Preseasonal | Allergovit® (Allergopharma, Rheinbeck, Germany) | - | 36 | 14.29 | 5 | SS, MS, SMS |
|  |  |  |  |  | PT |  | Standardized PT | 14 | 10/4 | 9.1 (7–16) |  |  |  | - |  | - |  |  | 28.57 |  |  |
| Valovirta | 2006 | Finland | DBRCT | Positive SPT ≥3, positive specific IgE (≥class 2), Positive CPT ≤ 100 000 SQ-U/ml, bronchial provocation test with methacholine PD_20≥_ 150 μg | SLIT | Random | Two doses of SLIT with a tree pollen extract; five times per week with a 5-week updosing phase and a maintenance period of up to 18 months;  Dose group 1: for the updosing-phase, tree pollen extract in the concentrations 150, 400, 1500, 4000 and 12 000 SQ-U/ml, for maintenance phase, a concentration of 12000 SQ-U/ml, the accumulated weekly dose corresponded to 24000 SQ-U or 3.6 lg major allergen Bet v 1/Aln g 1/Cor a 1;  Dose group 2: for the updosing phase, tree pollen extract in the concentrations 1500, 4000, 12000, 35000 and 100 000 SQ-U/ml and, for maintenance, a concentration of 100000 SQ-U/ml, the accumulated weekly dose corresponded to 200000 SQ-U or 30 lg major allergen Bet v 1/Aln g 1/Cor a 1 | 59 | 32/27 | 9.3±2.9 | 4.6±2.2 | Tree pollen | - | Drops | Continuous | Allergen extract of SQ standardized B. verrucosa (birch), C. avellana (hazel) and A. glutinosa (alder) | Conjunctivitis 57, asthma 26, atopic dermatitis 22 | 17 (10-20) | 9.23 | 5 | SS, MS, TRAE |
|  |  |  |  |  | Placebo |  | Placebo containing 50% glycerol and 50% saline buffer | 29 | 18/11 | 9.9±3.0 | 4.6±2.5 |  |  |  |  | - | Conjunctivitis 29, asthma 10, atopic dermatitis 5 |  | 12.12 |  |  |
| Novembre | 2004 | Italy | RCT | Clinical symptoms limited to the GPS, together with a positive grass extract SPT response (wheal diameter >3 mm) | SLIT+PT | Random | SLIT for 4 months + symptomatic drugs; during the build-up phase, received 2 daily administrations (morning and evening), starting with one drop from the most diluted vial and ending on day 15 with 5 drops from the most concentrated vial, the maintenance dose of 5 drops of the 25 BU/mL concentration (corresponding to 0.5 mg of the major grass allergen group 5) administered once daily in the morning 5 times a week (Monday to Friday) until the end of June, without any changes during the pollen season | 47 | 33/14 | 8.96 (5-14) | - | Grass pollen | Mono-sensitized | Drops | Coseasonal | An extract of mixed grass pollens (ALK-Abello`, Madrid, Spain) | - | 36 | 12.96 | 3 | Development of asthma |
|  |  |  |  |  | PT |  | Symptomatic drugs | 50 | 35/15 | 7.74 (4-16) |  |  |  | - |  | - |  |  | 15.25 |  |  |
| Rolinck-Werninghaus | 2004 | Germany | DBRCT | Specific IgE to grass pollen (CAP-class≥2), a wheal size of ≥3 mm or a skin index of ≥0.6 in SPT, and a positive CPT at a specific allergen concentration≤100 000 SQ-U/ml | SLIT | Random | Standardized allergens from a 5-grass mixture; during first 4 weeks, from five vials (0–4) with the concentrations 1.6, 8, 40, 200 and 1000 STU/ml beginning with one drop from vial 0 taken on days 1 and 2 and continued by two drops on days 2 and 3 until reaching five drops on days 9 and 10, then increased by 1 drop/day from one to five drops from vials 1–4, five drops from vial 4 applied as a maintenance dose three times a week until the end of the 32 months treatment period | 49 | 30/19 | 3-14 | - | Grass and/or rye pollen | - | Drops | Continuous | Pangramin SLIT® (ALK-SCHERAX, Hamburg, Germany). | Asthma 20, atopic dermatitis 11 | 32 | 22.45 | 3 | SS, MS, TRAE |
|  |  |  |  |  | Placebo |  | Placebo vials contained the Pangramin solution without allergen | 48 | 35/13 |  |  |  |  |  |  | - | Asthma 19, atopic dermatitis 6 |  | 22.92 |  |  |
| Eng | 2002 | Switzerland | Prospective cohort | IgE-mediated sensitivity to seasonal allergens only (grass pollen with or without tree pollen) as assessed by SPT and RAST | SCIT + PT | Parental preference | A standardized grass pollen depot-allergoid | 13 | 10/3 | 9.6 (5-16) | 3.8 | Grass pollen | - | Injection | Preseasonal | Allergovit® (Allergopharma, Rheinbek, Germany) | Asthma 9 | 36 | 7.14 | 5 | New sensitization |
|  |  |  |  |  | PT |  | Standardized PT only | 10 | 8/2 | 8.8 (7-13) | 3.3 |  |  | - |  | - | Asthma 8 |  | 28.57 |  |  |
| Cengizlier | 1999 | Turkey | DBRCT | History, physical examination findings, positive intradermal skin tests, and nasal provocation tests | SCIT | Random | Immunotherapy; the initial dose 0.1 mL twice weekly, gradually increased to 0.5 ml of 500 AU/mL solution as the maintenance dose, after maintained on this dose for the first 11 weeks, switched to weekly injections until the end of the 6-month study period | 19 | 9/10 | 10±6 | - | HDM | Mono-sensitized | Injection | - | - | - | 6 | 0 | 2 | SS |
|  |  |  |  |  | Non-SCIT |  | Without any treatment | 17 | 7/10 | 11±5 |  |  |  | - |  |  |  |  |  |  |  |
| La Rosa | 1999 | Italy | DBRCT | Positive history of rhinoconjunctivitis caused by Parietaria pollen sensitization documented by positive SPT (wheal diameter >4mm) and positive radioallergosorbent test results (class II and above) | SLIT | Random | A standardized P judaica extract; the build-up treatment phase started with 2 drops of the first concentration (1 IR/mL), increasing by 2 drops per day up to 10 drops per day, followed by the next concentration (10 IR/mL) increasing from 2 to 10 drops per day and then continued with a similar progression from 2 to 20 drops of the next concentration (100 IR/mL) and 2 to 20 drops of the highest concentration (300 IR/mL), after administration of 20 drops of 300 IR per milliliter daily for 1 month, the maintenance dose 20 drops of 300 IR per milliliter 3 times a week until the end of the study | 20 | 13/7 | 10 (6-14) | 3±2 | Grass pollen | - | Drops | Continuous | Standardized P judaica extract (Stallergènes, Antony, France) | - | 24 | 20 | 4 | TRAE |
|  |  |  |  |  | Placebo |  | Placebo consisted of a glycerinated phenolated saline solution with an appearance and taste similar to those of the active treatment | 21 | 12/9 | 10 (7-13) | 4±3 |  |  |  |  | - |  |  | 19.05 |  |  |
| Vourdas | 1998 | Greece | DBRCT | Sensitization to olive pollen proved by positive SPT and positive RAST class II and above | SLIT | Random | A standardized olive pollen extract; started with two drops of the lowest concentration (1 IR/ml), increasing by two drops per day up to a maximum of 10 drops/day, continued with two drops of the next concentration (10 IR/ml), increasing by two drops per day up to 10 drops/day, and then progressing in a similar way from two to 20 drops of the next concentration (100 IR/ml) and then of the highest concentration (300 IR/ml), the maintenance dose 20 drops of 300 IR/ml daily for 5 months, in July, the treatment stopped until the next January | 34 | 25/9 | 12 (8-17) | 4 (2-8) | Olive pollen | Mono-sensitized 4, poly-sensitized 30 | Drops | Pre-and coseasonal | Olive pollen extract (Stallergenes SA) | Asthma 33 | 12 | 2.94 | 5 | TRAE |
|  |  |  |  |  | Placebo |  | Placebo consisted of a glycerinated phenolated saline solution with an appearance similar to that of the active agent | 32 | 24/8 | 12 (7-17) | 4 (2-8) |  | Mono-sensitized 6, poly-sensitized 26 |  |  | - | Asthma 29 |  | 3.13 |  |  |

*Median (interquartile range).

AIT, allergen immunotherapy; AR, allergic rhinitis; DBRCT, double-blind randomized controlled trial; GPS, grass pollen season; HDM, house dust mites; ICS, inhaled corticosteroids; IgE, immunoglobulin E; PT, pharmacotherapy; M/F, male/female; QA, quality assessment; RAST, radioallergosorbent assay; RCT, randomized controlled trial; SABA, short-acting beta2-agonist; SCIT, subcutaneous immunotherapy; SLIT, sublingual immunotherapy; SS, system score; MS, medication score; SMS, system and medication score; TRAE, treatment-related adverse event; SPT, skin prick test; ARIA, Allergic Rhinitis and its Impact on Asthma.
