# Supplementary material for: Efficacy and safety of sublingual versus subcutaneous immunotherapy in children with allergic rhinitis: a systematic review and meta-analysis
Source: Front Immunol. 2023 Dec 15;14:1274241. doi: 10.3389/fimmu.2023.1274241 (PMC10757840; doi:10.3389/fimmu.2023.1274241)
Supplement: Supplementary file 13 [file Table_2.docx]

**Supplementary Table S2** Meta-regression analysis for the source of heterogeneity.

| **Group** | **Outcome** | **Variable** | **Coef** | **Std. Err** | | **t** | ***P*** | **95%CI** | |
| --- | --- | --- | --- | --- | --- | --- | --- | --- | --- |
| **SLIT vs non-SLIT** | | | | | | | | | |
|  | **SSs** |  |  |  |  | |  |  |  |
|  |  | Design (RCT vs. cohort) | -1.103 | 1.774 | -0.62 | | 0.541 | -4.782 | 2.577 |
|  |  | Allergen (pollen vs. HDM) | 2.146 | 1.318 | 1.63 | | 0.119 | -0.596 | 4.888 |
|  |  | Duration (12-23 vs. <12) | -1.783 | 1.921 | -0.93 | | 0.364 | -5.791 | 2.225 |
|  |  | Duration (≥24 vs. <12) | -1.877 | 1.682 | -1.12 | | 0.278 | -5.386 | 1.631 |
|  |  | Modality (tablets vs. drops) | 1.708 | 1.493 | 1.14 | | 0.265 | -1.389 | 4.805 |
|  |  | Protocol (pre-and co-seasonal vs. continuous) | 1.775 | 1.586 | 1.12 | | 0.276 | -1.533 | 5.083 |
|  |  | Protocol (after the GPS vs. continuous) | 2.119 | 2.465 | 0.86 | | 0.400 | -3.023 | 7.261 |
|  |  | Constant | -0.651 | 1.618 | -0.4 | | 0.691 | -4.007 | 2.704 |
|  | **MSs** |  |  |  |  | |  |  |  |
|  |  | Design (RCT vs. cohort) | 1.212 | 0.881 | 1.38 | | 0.187 | -0.647 | 3.072 |
|  |  | Allergen (pollen vs. HDM) | 1.329 | 0.642 | 2.07 | | 0.055 | -0.032 | 2.690 |
|  |  | Duration (12-23 vs. <12) | -1.193 | 0.842 | -1.42 | | 0.177 | -2.988 | 0.601 |
|  |  | Duration (≥24 vs. <12) | -1.118 | 0.798 | -1.40 | | 0.181 | -2.819 | 0.583 |
|  |  | Modality (tablets vs. drops) | 1.018 | 0.680 | 1.5 | | 0.153 | -0.417 | 2.453 |
|  |  | Protocol (pre-and co-seasonal vs. continuous) | 0.831 | 0.767 | 1.08 | | 0.296 | -0.804 | 2.467 |
|  |  | Protocol (after the GPS vs. continuous) | 1.126 | 1.541 | 0.73 | | 0.476 | -2.159 | 4.411 |
|  |  | Constant | -1.885 | 0.809 | -2.33 | | 0.032 | -3.591 | -0.179 |
|  | **SMSs** |  |  |  |  | |  |  |  |
|  |  | Allergen (pollen vs. HDM) | 0.256 | 0.550 | 0.47 | | 0.66 | -1.157 | 1.670 |
|  |  | Duration (12-23 vs. <12) | 0.057 | 0.123 | 0.47 | | 0.665 | -0.284 | 0.399 |
|  |  | Duration (≥24 vs. <12) | -1.075 | 0.198 | -5.43 | | 0.006 | -1.624 | -0.526 |
|  |  | Modality (tablets vs. drops) | 0.732 | 0.380 | 1.93 | | 0.112 | -0.245 | 1.710 |
|  |  | Protocol (pre-and co-seasonal vs. continuous) | 0.297 | 0.503 | 0.59 | | 0.581 | -0.996 | 1.589 |
|  |  | Constant | -0.879 | 0.460 | -1.91 | | 0.115 | -2.062 | 0.305 |
|  | **New sensitizations** |  |  |  |  | |  |  |  |
|  |  | Design (RCT vs. cohort) | -0.859 | 0.404 | -2.12 | | 0.168 | -2.599 | 0.881 |
|  |  | Duration (≥24 vs. 12-23) | 0.480 | 0.755 | 0.64 | | 0.59 | -2.770 | 3.729 |
|  |  | Constant | 0.970 | 0.200 | 4.85 | | 0.04 | 0.110 | 1.830 |
|  | **Development of asthma** |  |  |  |  | |  |  |  |
|  |  | Design (RCT vs. cohort) | 0.025 | 0.429 | 0.06 | | 0.959 | -1.821 | 1.871 |
|  |  | Modality (tablets vs. drops) | 0.391 | 0.320 | 1.22 | | 0.345 | -0.984 | 1.767 |
|  |  | Protocol (co-seasonal vs. continuous) | -0.131 | 0.469 | -0.28 | | 0.826 | -6.093 | 5.831 |
|  |  | Protocol (after the GPS vs. continuous) | 0.341 | 0.367 | 0.93 | | 0.523 | -4.322 | 5.004 |
|  |  | Constant | 0.663 | 0.365 | 1.81 | | 0.211 | -0.909 | 2.235 |
|  | **TRAEs** |  |  |  |  | |  |  |  |
|  |  | Allergen (pollen vs. HDM) | -0.274 | 0.497 | -0.55 | | 0.588 | -1.310 | 0.763 |
|  |  | Duration (12-23 vs. <12) | 0.539 | 0.550 | 0.98 | | 0.34 | -0.617 | 1.694 |
|  |  | Duration (≥24 vs. <12) | -1.017 | 0.604 | -1.68 | | 0.109 | -2.285 | 0.251 |
|  |  | Modality (tablets vs. drops) | 0.860 | 0.456 | 1.89 | | 0.074 | -0.091 | 1.811 |
|  |  | Protocol (pre-and co-seasonal vs. continuous) | 0.708 | 0.559 | 1.27 | | 0.221 | -0.466 | 1.883 |
|  |  | Protocol (after the GPS vs. continuous) | 0.095 | 1.138 | 0.08 | | 0.935 | -2.296 | 2.485 |
|  |  | Constant | 2.948 | 0.391 | 7.54 | | 0 | 2.133 | 3.763 |
| **SCIT vs non-SCIT** | | | | | | | | | |
|  | **SSs** |  |  |  |  | |  |  |  |
|  |  | Design (RCT vs. cohort) | 2.770 | 3.333 | 0.83 | | 0.467 | -7.837 | 13.376 |
|  |  | Allergen (pollen vs. HDM) | 2.304 | 3.469 | 0.66 | | 0.554 | -8.736 | 13.345 |
|  |  | Duration (12-23 vs. <12) | -1.336 | 4.894 | -0.27 | | 0.811 | -22.392 | 19.721 |
|  |  | Duration (≥24 vs. <12) | -3.284 | 4.013 | -0.82 | | 0.499 | -20.552 | 13.984 |
|  |  | Protocol (pre-seasonal vs. continuous) | -1.336 | 4.894 | -0.27 | | 0.811 | -22.392 | 19.721 |
|  |  | Constant | -3.310 | 1.498 | -2.21 | | 0.114 | -8.077 | 1.457 |
|  | **MSs** |  |  |  |  | |  |  |  |
|  |  | Allergen (pollen vs. HDM) | 0.647 | 2.126 | 0.30 | | 0.812 | -26.363 | 27.657 |
|  |  | Duration (≥24 vs. 12-23) | 2.201 | 0.302 | 7.28 | | 0.087 | -1.641 | 6.044 |
|  |  | Protocol (pre-seasonal vs. continuous) | 0.647 | 2.126 | 0.30 | | 0.812 | -26.363 | 27.657 |
|  |  | Constant | -1.620 | 1.207 | -1.34 | | 0.408 | -16.955 | 13.715 |
| **SLIT vs SCIT** | | | | | | | | | |
|  | **SSs** |  |  |  |  | |  |  |  |
|  |  | Design (RCT vs. cohort) | -0.624 | 0.946 | -0.66 | | 0.629 | -12.649 | 11.401 |
|  |  | Constant | 0.624 | 0.555 | 1.13 | | 0.463 | -6.423 | 7.671 |

SLIT, sublingual immunotherapy; SCIT, subcutaneous immunotherapy; CI, confidence interval; RCT, randomized controlled trial; GPS, grass pollen season; HDM, house dust mite; SSs, symptom scores; MSs, medication scores; SMSs, symptom and medication scores; TRAEs, treatment-related adverse events; Coef, coefficient; Std. Err, standard error.
